# Diagnostic Accuracy of PET for Differentiating True Glioma Progression From Post Treatment-Related Changes: A Systematic Review and Meta-Analysis

**DOI:** 10.3389/fneur.2021.671867

**Published:** 2021-05-20

**Authors:** Meng Cui, Rocío Isabel Zorrilla-Veloz, Jian Hu, Bing Guan, Xiaodong Ma

**Affiliations:** ^1^Medical School of Chinese People's Liberation Army, Beijing, China; ^2^Department of Neurosurgery, The First Medical Centre of Chinese People's Liberation Army General Hospital, Beijing, China; ^3^Department of Cancer Biology, The University of Texas MD Anderson Cancer Center, Houston, TX, United States; ^4^The University of Texas MD Anderson Cancer Centre UT Health Graduate School of Biomedical Sciences, Houston, TX, United States; ^5^Department of Health Economics, The First Medical Centre of Chinese People's Liberation Army General Hospital, Beijing, China

**Keywords:** glioma, positron emission tomography, glioma progression, treatment outcome, meta-analysis

## Abstract

**Purpose:** To evaluate the diagnostic accuracy of PET with different radiotracers and parameters in differentiating between true glioma progression (TPR) and post treatment-related change (PTRC).

**Methods:** Studies on using PET to differentiate between TPR and PTRC were screened from the PubMed and Embase databases. By following the PRISMA checklist, the quality assessment of included studies was performed, the true positive and negative values (TP and TN), false positive and negative values (FP and FN), and general characteristics of all the included studies were extracted. Results of PET consistent with reference standard were defined as TP or TN. The pooled sensitivity (Sen), specificity (Spe), and hierarchical summary receiver operating characteristic curves (HSROC) were generated to evaluate the diagnostic accuracy.

**Results:** The 33 included studies had 1,734 patients with 1,811 lesions suspected of glioma recurrence. Fifteen studies tested the accuracy of ^18^F-FET PET, 12 tested ^18^F-FDG PET, seven tested ^11^C-MET PET, and three tested ^18^F-DOPA PET. ^18^F-FET PET showed a pooled Sen and Spe of 0.88 (95% CI: 0.80, 0.93) and 0.78 (0.69, 0.85), respectively. In the subgroup analysis of FET-PET, diagnostic accuracy of high-grade gliomas (HGGs) was higher than that of mixed-grade gliomas (*P*_interaction_ = 0.04). ^18^F-FDG PET showed a pooled Sen and Spe of 0.78 (95% CI: 0.71, 0.83) and 0.87 (0.80, 0.92), the Spe of the HGGs group was lower than that of the low-grade gliomas group (0.82 vs. 0.90, *P* = 0.02). ^11^C-MET PET had a pooled Sen and Spe of 0.92 (95% CI: 0.83, 0.96) and 0.78 (0.69, 0.86). ^18^F-DOPA PET had a pooled Sen and Spe of 0.85 (95% CI: 0.80, 0.89) and 0.70 (0.60, 0.79). FET-PET combined with MRI had a pooled Sen and Spe of 0.88 (95% CI: 0.78, 0.94) and 0.76 (0.57, 0.88). Multi-parameters analysis of FET-PET had pooled Sen and Spe values of 0.88 (95% CI: 0.81, 0.92) and 0.79 (0.63, 0.89).

**Conclusion:** PET has a moderate diagnostic accuracy in differentiating between TPR and PTRC. The high Sen of amino acid PET and high Spe of FDG-PET suggest that the combination of commonly used FET-PET and FDG-PET may be more accurate and promising, especially for low-grade glioma.

## Introduction

Glioma is the most common primary brain tumor. It includes low-grade gliomas (LGGs) and high-grade gliomas (HGGs). The annual incidence of gliomas is approximately six cases per 100,000 individuals worldwide (1). Maximizing extent of resection (EOR) was demonstrated to be associated with improved outcomes, including progression-free survival and overall survival, by multiple retrospective analyses (2, 3). According to the NCCN guidelines, the standard treatment method for LGGs is surgical gross total resection combined with radiotherapy (RT) and adjuvant chemotherapy (CT) of temozolomide (TMZ) or PCV (procarbazine, lomustine, vincristine); for HGGs it is total resection followed by concurrent and adjuvant chemotherapy of TMZ or PCV (more than six cycles) (4–6).

Tumor progression monitoring is very important for patients with glioma after treatment. A confirmed diagnosis of tumor recurrence or progression guides surgeon and patients for the next treatment strategy. Progression and recurrence can be described as true glioma progression (TPR), while all treatment effects can (e.g., radiation necrosis and pseudoprogression) be described as post treatment-related change (PTRC). The wrong differentiation between TPR and PTRC will cause the wrong decision to be made by surgeon and patients, for example an unnecessary secondary surgery or the best surgery opportunity missed. Assessment of the glioma treatment response to differentiate between PTRC and TPR remains as much of a challenge as the treatment (7, 8).

At present, imaging follow-up was still the most common method to monitor TPR. Gadolinium-enhanced T1-weighted magnetic resonance imaging (G-T1-MRI) is often the choice for monitoring glioma patients' response to PTRC and TPR. However, this test often gets a false positive result because it only detects the disruption of the blood-brain barrier and not the tumor activity specifically. To overcome the limitations of G-T1-MRI, multimodal MRI has been proposed, including magnetic resonance spectroscopy (MRS), diffusion-weighted imaging (DWI), and perfusion-weighted imaging (PWI), among others (9, 10). Some previous meta-analyses had demonstrated the relative high diagnostic accuracy of MRS and PWI but low accuracy of DWI (11–13). However, these methods also sometimes caused false positive or false negative results because of their inability to provide metabolic information of lesions directly.

Due to the challenge to accurately determine brain tumor response by MRI both in daily practice and clinical trials, radiolabeled amino acid-positron emission tomography (PET) was proposed in the management of brain tumors by the RANO (Response Assessment in Neuro-Oncology) group (14). The radiotracers include O-(2-[^18^F] fluoroethyl)-L-tyrosine (^18^F-FET), ^18^F-fluorodeoxyglucose (^18^F-FDG), ^11^C-methionine (^11^C-MET), 3,4-dihydroxy-6-[18F]-fluoro-l-phenylalanine (^18^F-DOPA), and ^11^C-choline (^11^C-CHO), among others. PET of various tracers can visualize biological processes such as cell proliferation, membrane biosynthesis, glucose consumption, and uptake of amino acid analogs. Hence PET provides additional insight beyond MRI into the biology and treatment response of gliomas which may be used for non-invasive grading, differential diagnosis, delineation of tumor extent, surgical and radiotherapy treatment planning, post treatment surveillance, and prognostication (15). Many previous studies have demonstrated the usefulness of PET for the assessment of the treatment response of glioma (16, 17), yet a systematic review and meta-analysis of the diagnostic accuracy of PET using different radiotracers and parameters is lacking. Here, we conducted a systematic review and meta-analysis to assess the diagnostic accuracy of PET at differentiating TPR and PTRC.

## Methods

### Search Strategy

This systematic review and meta-analysis was performed according to the Preferred Reporting Items for Systematic Reviews and Meta-Analysis (PRISMA) criteria (Supplementary Material 1). It was registered on PROSPERO with registration number CRD42020197852. PubMed and Embase were searched using the key words as follows: “glioma” or “glioblastoma” or “astrocytoma” or “oligodendroglioma” or “oligoastrocytoma”; and “Positron Emission Tomography” or “PET;” and “progression” or “recurrence” or “recurrent” or “relapse” or “pseudoprogression” or “necrosis” or “posttreatment.” The detailed search strategy is shown in Supplementary Material 2.

### Selection Criteria

The inclusion criteria were as follows: English language, publication from January 1999 to December 2019, clinical diagnostic test for glioma with adequate PET data (radiotracers, imaging technique, parameters, etc.), more than 15 patients, adult patients with glioma treated with standard therapy (surgery+RT/CT), diagnostic test involving PET to differentiate between TPR and PTRC compared to a definite reference standard diagnosis (histology or clinical/imaging follow-up), and 2 × 2 tables from which true positives (TP), false positives (FP), false negatives (FN), and true negatives (TN) could be extracted. The reference standard of clinical/imaging follow-up was reasonable according to RANO criteria. For WHO grade II gliomas, the PTRC required that both the clinical and the radiological situation had to be stable/improved for at least 12 months without administration of another therapy. For WHO grade III-IV gliomas, the classification of PTRC required at least 6 months of stable or improved clinical and radiological condition, as well as no change in tumor treatment. TPR was considered present when lesions continued to increase in size on at least two subsequent MRI scans, paralleled by a deterioration in performance status, or when a patient died of glioma, which ever occurred first (18).

Exclusion criteria were the inclusion of other tumor entities besides glioma (such as brain metastases, lymphomas, or meningiomas); pediatric glioma patients; *in vitro* or animal studies; publication as a review, letter, comment, case report, or abstract; and treatment methods other than standard therapy recommended by NCCN guidelines (6), such as antiangiogenic bevacizumab therapy, intracavitary radiation, and tumor-treating field therapy.

### Data Extraction and Quality Assessment

After duplicates were eliminated, studies were screened for eligibility based first on their title and abstract and subsequently on their full text independently by a board-certified neurosurgeon with 6 years of experience and a neuro-oncologist with 5 years of experience. Study quality was assessed independently according to the quality assessment of diagnostic accuracy studies (QUADAS-2) (19). Then these two authors reviewed in detail the abstracts, methods, results, figures, and tables. Extracted data consisted of true positives (TP), false positives (FP), true negatives (TN), and false negatives (FN); if raw numbers were not reported in the papers, we calculated them from the sensitivity (Sen) and specificity (Spe). The general characteristics extracted from each study were the total number of patients, study design, patient selection criteria, therapy method, mean or median age, sex, reference standard of diagnosis (histopathology or clinical/MRI follow-up), time point at which tumor progression was suspected, PET characteristics and time at which PET was performed, and parameters of PET with or without cut-off value. Disagreements were reassessed by the two authors together to reach a consensus.

### Statistical Analysis

By using Meta-Disc statistical software version 1.4, we first evaluated the heterogeneity between each study caused by the threshold effect. The Spearman correlation coefficient between the logit of Sen and the logit of (1–Spe) was computed to assess the threshold effect (20). A strong positive correlation would suggest a threshold effect with *P* < 0.05.

Then, we used Stata 14.0 to perform the statistical analysis: (1) To evaluate the extent of heterogeneity in each study, we used the Q test and the inconsistency index (*I*^2^) of the diagnostic odds ratio (DOR: the ratio of LR+ and LR-). Heterogeneity was considered to be significant if *P* < 0.1 or *I*^2^ > 50%. In this case, the Sen and Spe of the studies were pooled using a random-effects model. Otherwise, a fixed-effects model was used (21). (2) The pooled Sen, Spe, and DOR with their 95% confidence intervals (CIs) were calculated for all studies and are presented as forest plots. If any cells of studies contained a count of zero, a value of one was added to replace zero in order to avoid any impossibilities in odds calculations for studies with a Sen or Spe of 100%. (3) The hierarchical logistic regression model was used to generate hierarchical summary receiver operating characteristic (HSROC) curves and area under the curve (AUC) to evaluate the diagnostic accuracy (22–24). (4) We assessed publication bias visually by using a scatter plot (X axis was log DOR, Y axis was ESS^1/2^, which is the inverse of the square root of effective sample size). A symmetric funnel shape and *P* > 0.01 in Deeks' asymmetry test indicated the absence of publication bias; otherwise, there was significant bias.

Subgroup analysis was performed using meta-regression if ≥3 studies could be included, and meta-regression tests were used to analyze differences between subgroups.

## Results

### Characteristics of Included Studies

**Figure 1:**
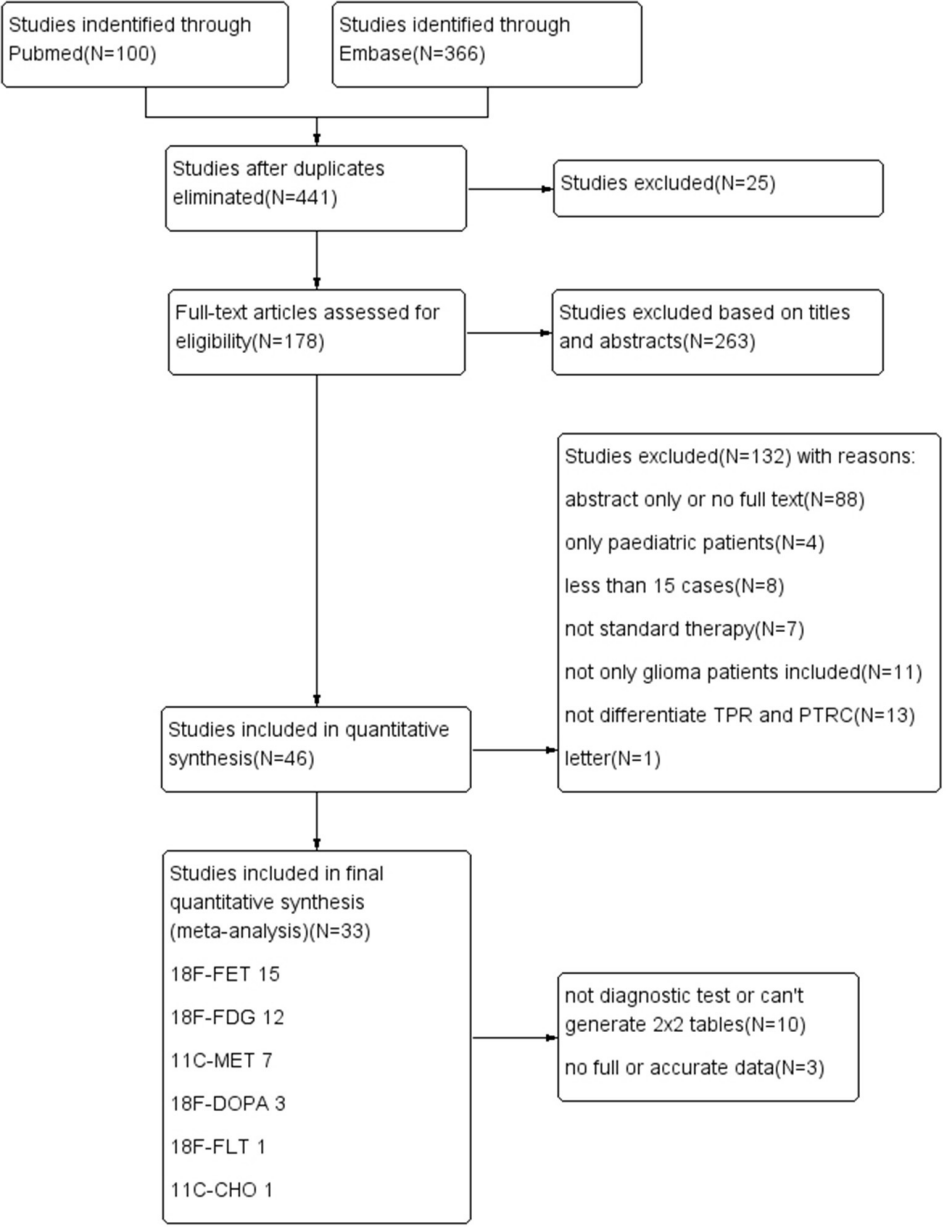
Flow diagram of the study-screening process.

**Figure 2:**
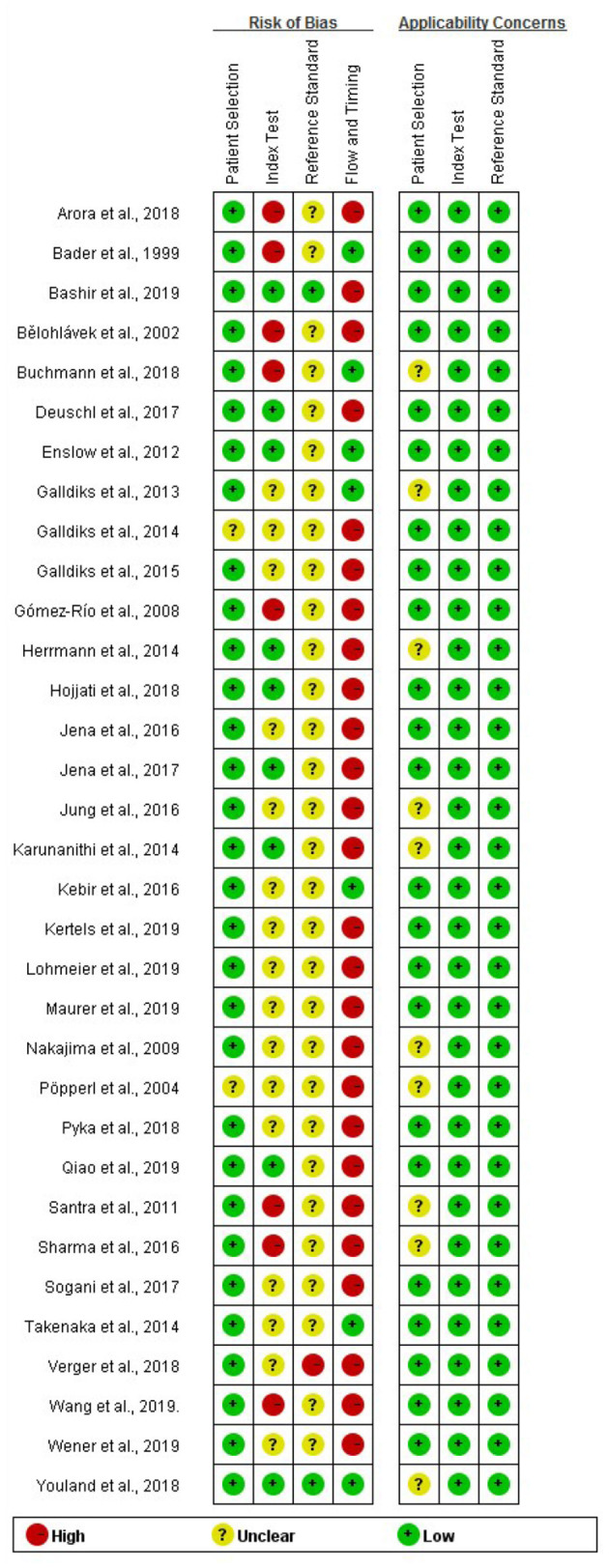
Methodological quality assessment summary of the included studies.

A flow diagram describing our study search process is provided in Figure 1. The results of the quality assessment are presented in Supplementary Material 3 and Figure 2. In brief, the quality of the included studies was satisfactory.

In total, the 33 included studies (retrospective = 21, prospective = 12) consisted of 1,734 patients and 1,811 lesions suspected of glioma recurrence. The mean ages ranged from 33.9 to 59.5 years. There were 1,110 males and 624 females. The lesions comprised 388 (21.4%) LGGs (WHO grades I and II) and 1,300 (71.8%) HGGs (WHO grades III and IV). In addition, 16 (0.9%) were other kinds of brain tumors, and 107 (5.9%) pathology results of lesions were not mentioned or determined.

Among the included studies, 15 tested the accuracy of ^18^F-FET PET (25–39), 12 tested ^18^F-FDG PET (40–51), seven tested ^11^C-MET PET (46, 47, 51–55), three tested ^18^F-DOPA PET (45, 56, 57), one tested ^18^F-FLT PET (44), and 1 tested ^11^C-CHO PET (46). Of all the included lesions, 836 (46.0%) had histopathology as the reference standard, 549 (30.2%) had clinical combined with imaging follow-up, and 81 (4.5%) only had imaging or clinical follow-up. Though the remaining 353 (19.4%) cases had either a combination or individual use of histopathology and clinical and imaging follow-up as the reference standard, the detailed data were not reported in these four studies (43, 47, 49, 51). The total incidence of TPR was 77.4% (1,394/1,801) in all patients. The incidence of TPR in HGG and LGG patients was 80.5% (577/717) and 63.1% (106/168), respectively. The incidence of PTRC was 22.6% (407/1,801) among all patients, 19.5% (140/717) in patients with HGG, and 36.9% (62/168) in patients with LGG (Supplementary Material 4).

### Quantitative Synthesis

**Figure 3:**
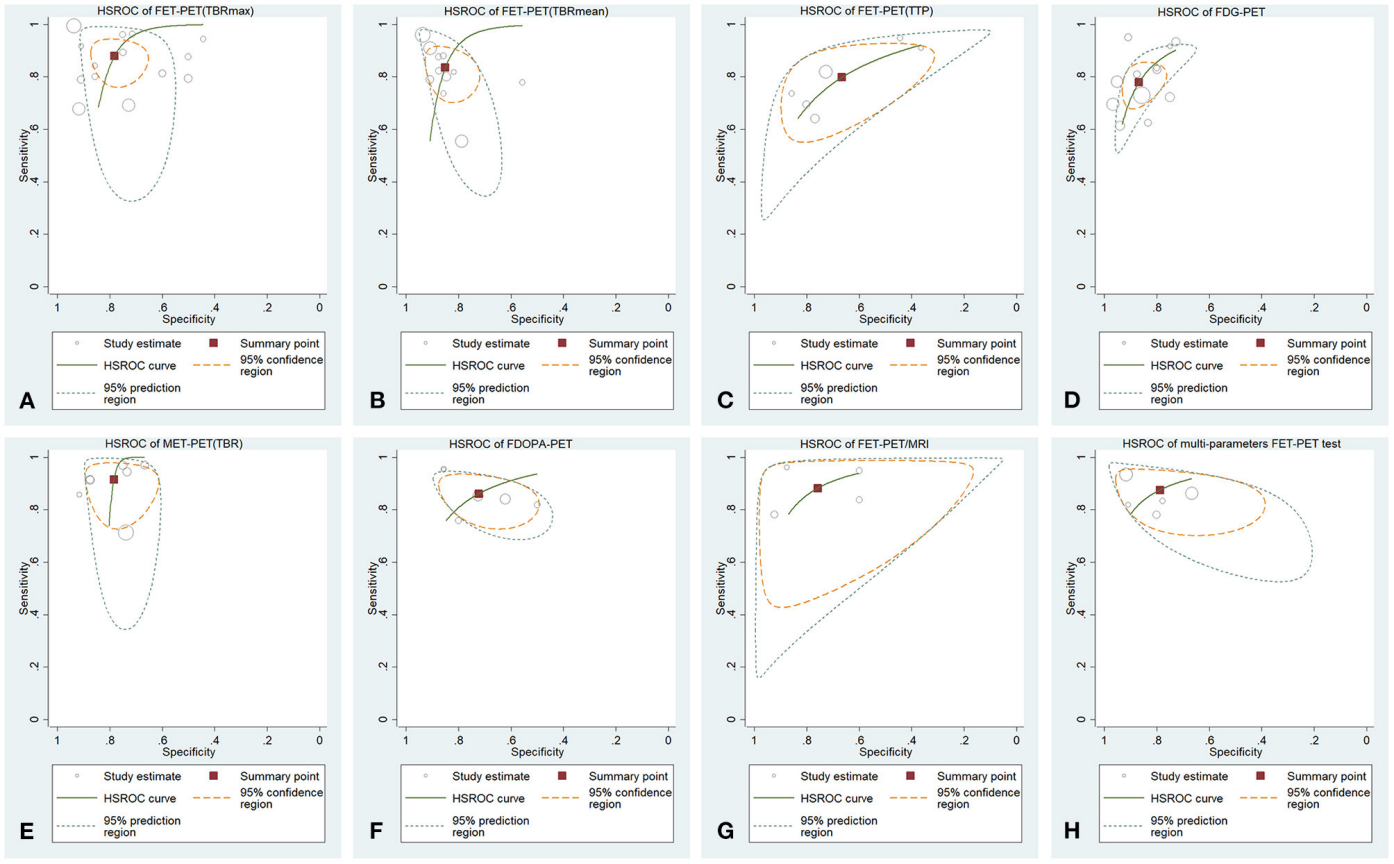
HSROC curves for **(A)** ^18^F-FET PET (TBRmax), **(B)** ^18^F-FET PET (TBRmean), **(C)** ^18^F-FET PET (TTP), **(D)** ^18^F-FDG PET, **(E)** ^11^C-MET PET (TBR), **(F)** ^18^F-DOPA PET, **(G)** FET-PET/MRI, and **(H)** multi-parameters FET-PET.

**Figure 4:**
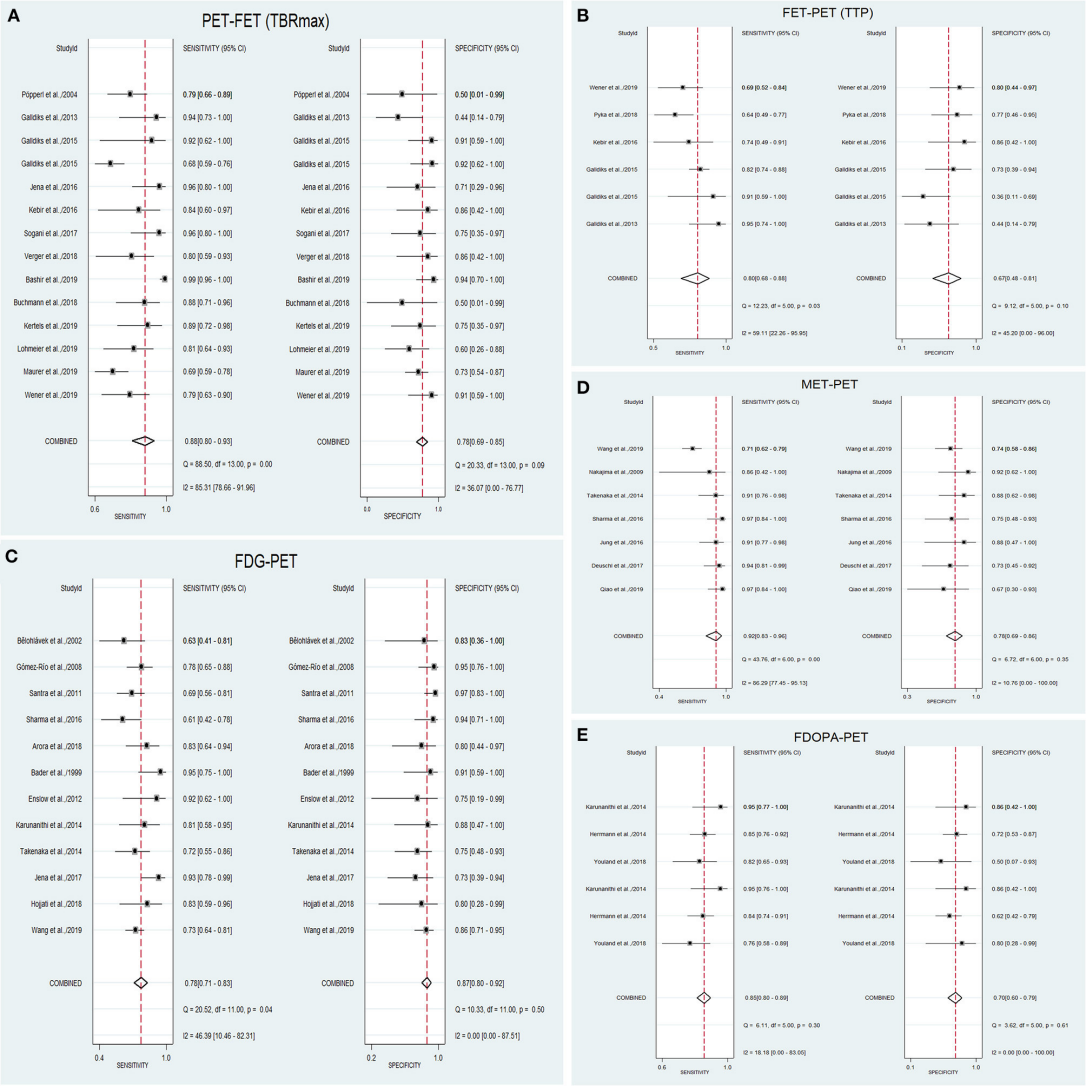
Forest plots for **(A)** ^18^F-FET PET (TBRmax), **(B)** ^18^F-FET PET (TTP), **(C)** ^18^F-FDG PET, **(D)** ^11^C-MET PET (TBR), and **(E)** ^18^F-DOPA PET.

The pooled results and subgroup analysis are shown in Tables 1, 2, respectively. The HSROC curves are shown in Figure 3. The forest plots are shown in Figure 4.

**Table 1:** Pooled Sen, Spe, DOR, and heterogeneity analysis results of PET tests of different radiotracers and techniques.

**Radiotracer and test technique**	**Quantitative parameter**	**Threshold range**	**ρ and P value**	**Heterogeneity of pooled Sen (upper) and Spe (lower) (*P*-value of Q test and *I*^**2**^)**	**Pooled Sen and its 95%CI**	**Pooled Spe and its 95%CI**	**Pooled DOR and its 95%CI**	**AUC of HSROC**
^18^F-FET	TBR_max_ (810 tests)	1.95,3.52	0.068 (*P* = 0.816)	**§*****P*** **<** **0.1**, ***I***^**2**^ **=** **85.3%**||*P* = 0.09, *I*^2^ = 36.1%	0.88 (0.80,0.93)	0.78 (0.69,0.85)	26 (12,57)	0.86 (0.83, 0.89)
	TBR_mean_ (713 tests)	1.52,2.98	**−0.677 (*****P*** **=** **0.022)**	NA*	NA*	NA*	NA*	0.90 (0.87, 0.92)
	TTP (317 tests)	20,45	0.714 (*P* = 0.111)	**§*****P*** **=** **0.03**, ***I***^**2**^ **=** **59.1%**¶*P* = 0.1, *I*^2^ = 45.2%	0.80 (0.68,0.88)	0.67 (0.48,0.81)	8 (4,16)	0.81 (0.77, 0.84)
^18^F-FDG (631 tests)	NA†	NA†	0.432 (*P* = 0.161)	—||*P* = 0.04, *I*^2^ = 46.4%¶*P* = 0.5, *I*^2^ = 0.0%	0.78 (0.71,0.83)	0.87 (0.80,0.92)	23 (14,39)	0.90 (0.87, 0.92)
^11^C-MET	TBR (409 tests)	1.43,2.51	0.559 (*P* = 0.192)	**§*****P*** **<** **0.1**, ***I***^**2**^ **=** **86.3%**¶*P* = 0.35, *I*^2^ = 10.8%	0.92 (0.83,0.96)	0.78 (0.69,0.86)	39 (15,105)	0.82 (0.78, 0.85)
^18^F-DOPA	TBR_max_ (175 tests), visual (175 tests)	NA†	−0.638 (*P* = 0.173)	¶*P* = 0.30, *I*^2^ = 18.2%¶*P* = 0.61, *I*^2^ = 0.0%	0.85 (0.80,0.89)	0.70 (0.60,0.79)	13 (7,24)	0.85 (0.82, 0.88)
FET-PET and MRI (190 tests)	NA‡	NA‡	0.316 (*P* = 0.648)	**§*****P*** **=** **0.06**, ***I***^**2**^ **=** **60.2%**¶*P* = 0.16, *I*^2^ = 42.5%	0.88 (0.78,0.94)	0.76 (0.57,0.88)	23 (9,59)	0.90 (0.87, 0.92)
FET-PET static/dynamic multi-parameters analysis (354 tests)	NA‡	NA‡	−0.100 (*P* = 0.873)	||*P* = 0.09, *I*^2^ = 49.5%¶*P* = 0.30, *I*^2^ = 18.4%	0.88 (0.81,0.92)	0.79 (0.63,0.89)	26 (9,78)	0.91 (0.88, 0.93)

AUC, area under receiver operating curve; CI, confidence interval; DOR, diagnostic odds ratio; HSROC, hierarchical summary receiver operating characteristic; NA^*^, not available because of the threshold effect existed; NA†, not available because not all the studies used quantitative parameter analysis of PET; NA‡, not available because different parameters were used among studies; ρ, Spearman correlation coefficient; TBR, tumor-to-brain ratio; TTP, time-to-peak;

§ *, significant heterogeneity*;

|| *, slight heterogeneity*;

¶ *, no heterogeneity*;

*#, some studies only reported the value of TBR, so it was used instead of TBRmax or TBRmean to analyze the pooled effect. Bold face type indicates statistical significance*.

**Table 2:** Subgroup analysis between LGGs, mixed-grade gliomas, and HGGs; between static PET scan and dynamic PET scan; and between visual assessment of the PET scan and semi-quantitative parametric analysis.

**Radiotracer and parameter**	**Subgroup**	**Number of studies/PET scan tests**	**Pooled Sen and its 95%CI**	***P*-value**	**Pooled Spe and its 95%CI**	***P*-value**	**Pooled DOR and its 95%CI**	**AUC**	$P_{\text{INTERACTION}}^{*}$
^18^F-FET, TBR_max_	HGGs	6/333	0.92 (0.85,0.98)	0.51	0.88 (0.79,0.96)	0.90	75 (18,304)	0.92	**0.04**
	Gliomas of mixed grades	8/477	0.84 (0.75,0.93)		0.71 (0.61,0.81)		12 (6,23)	0.80	
	Static scan	5/311	0.94 (0.88,0.99)	0.80	0.76 (0.62,0.90)	0.09	47 (7,299)	0.92	0.10
	Dynamic scan	9/810	0.82 (0.73,0.91)		0.79 (0.71,0.88)		15 (7,32)	0.86	
	PET performed ≤7d after SRR	3/108	0.84 (0.76,0.92)	0.19	0.79 (0.56,1.00)	0.64	NA	NA	0.34
	PET performed >7d after SRR	4/233	0.76 (0.68,0.84)		0.82 (0.68,0.95)		NA	NA	
^18^F-FDG	Visual assessment	8/355	0.76 (0.63,0.85)	0.30	0.90 (0.81,0.95)	**<0.05**	29 (14,60)	0.92	0.17
	Semi-quantitative parametric analysis	7/349	0.83 (0.73,0.90)		0.82 (0.72,0.89)		23 (10,51)	0.85	
	HGGs	11/214	0.76 (0.68,0.83)		0.82 (0.70,0.90)		15 (7,33)	0.84	
	Gliomas of mixed grades	7/417	0.76 (0.58,0.88)	0.31†	0.87 (0.80,0.92)	**<0.05**†	21 (8,52)	0.88	0.64†
	LGGs	5/141	0.61 (0.34,0.83)	0.83†	0.90 (0.77,0.96)	**0.02**†	15 (3,88)	0.90	0.10†
^11^C-MET, TBR	HGGs	3/248	0.94 (0.87,1.00)	0.95	0.82 (0.69,0.95)	0.47	71 (20,251)	0.95	0.58
	Gliomas of mixed grades	4/384	0.89 (0.78,0.99)		0.77 (0.67,0.86)		29 (7, 117)	0.79	
	PET performed <20 m after RT	2/92	0.96 (0.91,1.00)	0.89	0.71 (0.53,0.89)	**0.04**	NA	NA	0.11
	PET performed >20 m after RT	4/110	091 (0.84,0.97)		0.89 (0.79,0.99)		NA	NA	
^18^F-DOPA	Visual assessment	3/175	0.86 (0.80,0.92)	**<0.05**	0.72 (0.59,0.86)	0.22	16 (4,62)	0.93	0.8
	Semi-quantitative analysis of TBR_max_	3/175	0.84 (0.77,0.90)		0.68 (0.54,0.83)		14 (4,50)	0.85	

AUC, area under receiver operating curve; CI, confidence interval; DOR, diagnostic odds ratio; HGGs, high-grade gliomas; LGGs, low-grade gliomas; SRR, suspicious recurrence of glioma; TBR, tumor-to-brain ratio. Bold face type indicates statistical significance. *Based on meta regression.

† *Compared with group of HGGs*.

#### ^18^F-FET PET (TBRmax, TBRmean, TTP)

The pooled weighted values of tumor-to-brain maximum ratio (TBRmax) tests were Sen = 0.88 (95% CI: 0.80, 0.93), Spe = 0.78 (0.69, 0.85), DOR = 26 (12, 57), and AUC of HSROC curve = 0.86 (0.83, 0.89). The Sen of these studies had high heterogeneity (*P* < 0.1, *I*^2^ = 85.3%). Subgroup analysis of glioma histopathology showed that the HGG subgroup of the FET-PET test group benefitted from higher accuracy than the mixed-grade group (*P*_interaction_ = 0.04). Subgroup analysis of PET scans (static and dynamic scan groups) showed no significant difference in diagnostic accuracy (*P*_interaction_ = 0.54). Different time points of PET scans may influence the diagnostic accuracy, but subgroup analysis indicated no difference between the ≤7-day and >7-day groups after suspicious recurrence.

Thresholds of the tumor-to-brain mean ratio (TBRmean) ranged from 1.52 to 2.98. The Spearman's correlation coefficient (ρ) was −0.677 (*P* = 0.022), which indicated that a threshold effect was detected only in this group when quantitative synthesis was performed. Therefore, the Sen and Spe of studies could not be pooled directly. The AUC of the HSROC curve was 0.90 (0.87, 0.92), which was used to evaluate the diagnostic accuracy of the TBRmean tests of FET-PET.

The pooled weighted values of time-to-peak (TTP) tests were Sen = 0.80 (95% CI: 0.68, 0.88), Spe = 0.67 (0.48, 0.81), DOR = 8 (4, 16), and AUC = 0.81 (0.77, 0.84).

#### ^18^F-FDG PET

The pooled weighted values were Sen = 0.78 (95% CI: 0.71, 0.83), Spe = 0.87 (0.80, 0.92), and DOR = 23 (14, 39). The AUC of the HSROC curve was 0.90 (0.87, 0.92). Subgroup analysis of PET visual assessment and semiquantitative analysis of one parameter (such as TBR and SUV) were performed. The difference in Spe between these two groups was statistically significant (*P* < 0.05). Subgroup analysis of glioma histopathology showed that the mixed-grade group and LGG group had higher pooled Spe than the HGG group according to the FDG-PET test (0.87 vs. 0.82 [*P* < 0.05], 0.90 vs. 0.82 [*P* = 0.02]). However, the pooled Sen of the HGG group was not significantly different from those of the other two groups (both *P* > 0.05).

#### ^11^C-MET PET (TBR)

Heterogeneity was detected in pooled Sen (*P* < 0.1, *I*^2^ = 86.3%). The pooled Sen, Spe, and DOR were 0.92 (95% CI: 0.83, 0.96), 0.78 (0.69, 0.86), and 39 (15, 105), respectively. The AUC of the HSROC curve was 0.82 (0.78, 0.85). The subgroup of HGGs and the subgroup of mixed grades were analyzed, but no significant differences in diagnostic accuracy were detected (all *P* > 0.05). Subgroup analysis of time points showed a higher pooled Spe of the >20 months after radiotherapy group than the <20 months group (*P* = 0.04), but the diagnostic accuracy in these two groups was not different (*P*_interaction_ = 0.11).

#### ^18^F-DOPA PET (TBRmax, Visual)

The pooled values of Sen, Spe, and DOR were 0.85 (95% CI: 0.80, 0.89), 0.70 (0.60, 0.79), and 13 (7, 24), respectively. The AUC of the HSROC curve was 0.85 (0.82, 0.88). Subgroup analysis showed that there were significant differences between the visual and semiquantitative groups regarding sensitivity (*P* < 0.05).

### Publication Bias Assessment

**Figure 5:**
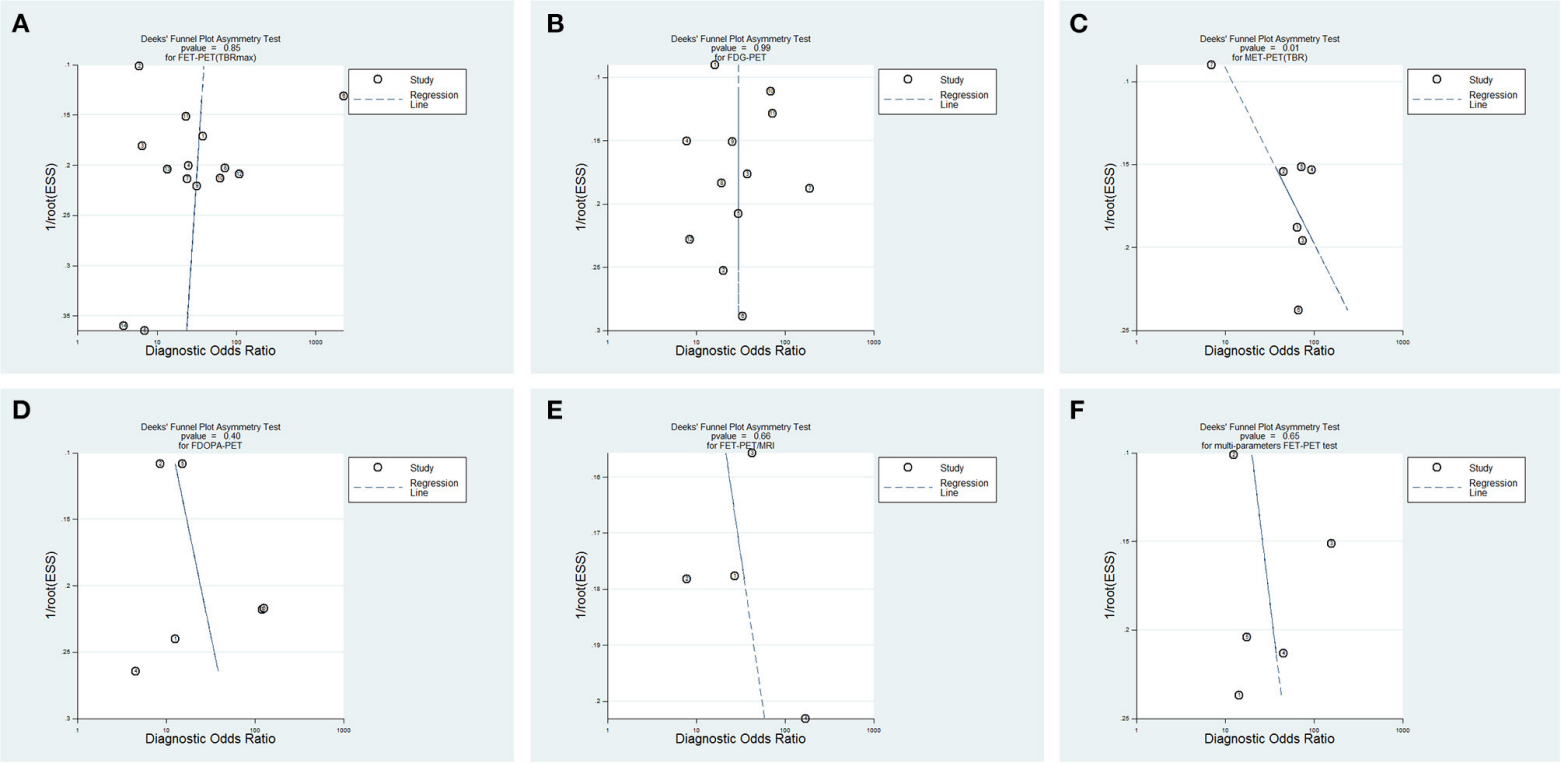
Funnel plots of publication bias for different PET tests. **(A)** ^18^F-FET PET (TBRmax), **(B)** ^18^F-FDG PET, **(C)** ^11^C-MET PET (TBR), **(D)** ^18^F-DOPA PET, **(E)** FET-PET/MRI, and **(F)** multi-parameters FET-PET.

Publication bias did not exist in the FET-PET (TBRmax), FDG-PET, or ^18^F-DOPA-PET studies (*P* = 0.85, *P* = 0.99, *P* = 0.40), while bias may have existed in the MET-PET (TBR) studies (*P* = 0.01) (Figure 5).

### MRI Combined With FET-PET and Multi-Parameter FET-PET

The pooled Sen, Spe, and DOR of the FET-PET/MRI group were 0.88 (95% CI: 0.78, 0.94), 0.76 (0.57, 0.88), and 23 (9, 59), respectively. The AUC of the HSROC curve was 0.90 (0.87, 0.92). The multi-parameter FET-PET test had pooled Sen, Spe, DOR, and AUC values of 0.88 (95% CI: 0.81, 0.92), 0.79 (0.63, 0.89), 26 (9, 78), and 0.91 (0.88, 0.93), respectively. Publication bias did not exist between these studies (*P* = 0.66, *P* = 0.65).

## Discussion

To overcome the limitations of MRI, PET-CT has been suggested by RANO for the management of glioma in addition to conventional MRI at every disease stage (58). Many studies have explored the diagnostic accuracy of PET labeled by different radiotracers, which indicates its potential value. Static and dynamic PET scans can acquire different parameters to assess diagnosis. Static scans can calculate SUVmax, SUVmean, TBRmax, and TBRmean to analyze the characteristics of radiotracer uptake in lesions and unaffected brain tissue to differentiate between tumor and normal brain tissue. Dynamic scanning brings in the variable of time and can depict the time-activity curves (TACs) of radiotracer uptake by lesions, and can then identify tumors by calculating TTP or slope (the change in SUV per hour) or by visually assessing the shape of TACs (28, 30, 39). All these parameters are believed to improve the diagnostic accuracy of PET tests compared to visual assessments alone.

According to the pooled AUC of HSROC curves in this meta-analysis, the diagnostic accuracy of ^18^FET-PET was similar to that of ^18^F-FDG PET, which was higher than that of ^18^F-DOPA PET and ^11^C-MET PET.

^18^F-FET PET had a high diagnostic accuracy for TBR. Subgroup analysis demonstrated a higher test accuracy for HGGs than gliomas of mixed grades (*P*_interaction_ = 0.04). Because of the higher recurrence rate of HGGs than LGGs, FET-PET will benefit HGG patients more during monitoring recurrence after surgery (Table 2). Compared with dynamic scanning, static FET-PET scanning may have similar accuracy for the differential diagnosis between TPR and PTRC (*P*_interaction_ = 0.10).

A common PET modality for the central nervous system (53, 59), ^11^C-MET PET had good test accuracy when calculating TBR (pooled set: 0.92, Spe: 0.78). Although the accuracy of ^11^C-MET PET was not different between the HGG group and the mixed-grade group (*P*_interaction_ = 0.58), a trend of higher diagnostic accuracy in the HGG group was detected. As seen for FET-PET, this result also indicated a better performance of MET-PET for HGG patients. ^18^F-DOPA is a kind of amino acid radiotracer that can be transported across the intact blood-brain barrier, but it is scarcely used or researched (60). The accuracy of 18F-DOPA PET was not superior to that of FET- or MET-PET in our analysis, which will limit the use of FDOPA-PET.

Because of the limited number of studies that reported the time when PET was performed, only the FET-PET TBRmax and MET-PET TBR could be evaluated for the influence of time point by subgroup analysis in this review. Because of the pseudoprogression caused by radiotherapy, the symptoms of patients and MRI always showed false positive results, which can be falsely considered as tumor recurrence. In our analysis, a longer time after radiotherapy showed a higher Spe of MET-PET, which indicated that the PET scan may need to be performed in the appropriate time window (perhaps a longer time after radiotherapy). Further research is needed to identify the best time point for performing PET after there is suspicious recurrence on MRI.

^18^F-FDG is a common radiotracer used in PET scans, but it always fails to differentiate between tumors and the brain due to the similarly high uptake rates of glucose in normal brain tissue (61). In this review, the accuracy of FDG-PET showed a similar accuracy to amino acid PET (Table 1). Compared to amino acid PET, FDG-PET had a higher Spe but a lower Sen, so we can predict that the combination of these two radiotracers would acquire better diagnostic performance. In the subgroup analysis (Table 2), the pooled Spe of visual assessment was higher than that of semiquantitative parametric analysis (*P* < 0.05). Compared to the mixed-grade glioma group and LGG group, the HGG group had a lower Spe (*P* < 0.05, *P* = 0.02). These results demonstrate that the high Spe of FDG-PET may be derived from the visual assessment of PET and the testing of LGG patients. Thus, visual assessment should also be an important part of PET tests in addition to parametric analysis. For HGG patients, the accuracy of amino acid PET was satisfactory, while for postoperative recurrence tests of LGG patients, amino acid PET was not good enough and should be combined with FDG-PET to acquire a higher Spe.

A confirmed diagnosis of recurrence during the follow-up of glioma patients is most important for choosing the next treatment method (secondary surgery or others). MRI is the most common and valuable method to monitor recurrence at present. From a review of some previous meta-analyses, the diagnostic accuracy of anatomical G-T1-MRI and advanced MRI is summarized in Table 3. The accuracy of G-T1-MRI and DWI was obviously not adequate for the diagnosis of glioma recurrence (10, 13). Advanced MRI showed a higher accuracy (11, 12, 62–64). Compared to advanced MRI, PET with different radiotracers did not present an obvious superiority in the differential diagnosis of glioma recurrence (Table 1). However, the high Sen of amino acid PET and high Spe of FDG-PET may suggest their combination in future applications of diagnosis.

**Table 3:** Previous meta-analysis of MRI accuracy for diagnosis of glioma recurrence.

**MRI**	**Study**	**Pooled Sen and its 95%CI**	**Pooled Spe and its 95%CI**	**DOR and its 95%CI**	**AUC**
Anatomical G-T1-MRI	(13)	0.68 (0.51,0.81)	0.77 (0.45,0.93)	NR	NR
	(64)	0.48 (0.08,0.90)	0.85 (0.39,0.98)	NR	NR
ADC of DWI	(13)	0.71 (0.60,0.80)	0.87 (0.77,0.93)	NR	NR
MRS	(63)	0.87 (0.80,0.92)	0.86 (0.77,0.93)	NR	0.93
	(13)	0.91 (0.79,0.97)	0.95 (0.65,0.99)	NR	NR
	(64)	0.82 (0.68,0.90)	0.79 (0.69,0.87)	NR	NR
Cho/NAA of MRS	(11)	0.88 (0.81,0.93)	0.86 (0.76,0.93)	37 (12,84)	0.92
Cho/Cr of MRS	(11)	0.83 (0.77,0.89)	0.83 (0.74,0.90)	24 (12,49)	0.90
DSC of PWI	(62)	0.88 (0.82,0.93)	0.85 (0.75,0.92)	42 (19,94)	0.93
	(12)	0.90 (0.85,0.94)	0.88 (0.83,0.92)	NR	NR
	(13)	0.87 (0.82,0.91)	0.86 (0.77,0.91)	NR	NR
DCE of PWI	(12)	0.89 (0.78,0.96)	0.85 (0.77,0.91)	NR	NR
	(13)	0.92 (0.73,0.98)	0.85 (0.76,0.92)	NR	NR

*ADC, apparent diffusion coefficient; Cho/Cr, choline/creatine; Cho/NAA, choline/N-acetyl-aspartate; DCE, dynamic contrast-enhanced; DSC, dynamic susceptibility contrast; DWI, diffusion-weighted imaging; G-T1-MRI, gadolinium-enhanced T1-weighted magnetic resonance; MRS, magnetic resonance spectroscopy; PWI, perfusion-weighted imaging; NR, not reported*.

Some previous meta-analyses evaluated the accuracy of PET, which did not show an obviously better performance than advanced MRI. Moreover, none of them took the different parameters (TBR, TTP, etc.) of PET into consideration (63–67). Along with the evaluation of PET with different tracers, this meta-analysis concentrated on the different accuracy of parameters of PET scans and showed more accuracy in the static parameter TBRmax of FET-PET than the dynamic parameter TTP. However, most studies in this review only performed static parameter analysis, and the accuracy of dynamic parameter analysis needs more research for evaluation. Regardless of these results, as a supplement to MRI, PET with different tracers and parameters can provide additional useful information by evaluating different metabolic pathways.

Furthermore, the accuracy of FET-PET multi-parameter analysis or FET-PET/MRI in this meta-analysis was similar to that of FET-PET TBR alone (Table 1). FET-PET multi-parameter analysis and hybrid PET/MRI also were not more accurate than advanced MRI. Although hybrid PET/MRI seemed to be a promising technique for recurrent glioma diagnosis, quantitative meta-analyses are scarce, and its accuracy in our analysis was not satisfactory (7, 68). PET-CT was also commonly used to differentiate TPR and PTRC in patients with brain metastases. A meta-analysis showed that pooled Sen and Spe of FDG-PET were 0.83 (95% CI: 0.74, 0.92) and 0.88 (0.81, 0.95), respectively. The pooled Sen and Spe of amino acid PET were 0.84 (0.79, 0.90) and 0.85 (0.80, 0.91) (69). These results were similar to the results in this review, thus it demonstrated similar diagnostic accuracy of PET differentiating TPR and PTRC both in glioma and brain metastases.

To acquire high diagnostic accuracy in the management of glioma as well as high feasibility and low cost to patients, the method of radiomics analysis is applied broadly now. PET radiomics combines datasets of static and dynamic PET parameters to make full use of imaging information. Precise image segmentation and feature extraction using different classification methods make it more accuracy in diagnosis. However, many studies demonstrated that a PET radiomics model had higher accuracy than the PET single parameter analysis in differentiating TPR from PTRC (70, 71). Feature-based PET/MRI radiomics also showed higher accuracy (72). One study used FDG PET, MET PET, and structural MRI images to develop an integrated radiomics-based model which showed a very high diagnostic accuracy with an AUC of 0.99 (51). In this review we did not cover radiomics. Multi-parameter PET radiomics combined with other imaging modalities has indeed great potential in differentiating TRP and PTRC, and its diagnostic performance should be summarized and evaluated in future.

Some limitations exist in this meta-analysis. First, while 33 studies were included, only three tested ^18^F-DOPA PET, one tested ^18^F-FLT PET, and one tested ^11^C-CHO PET. So their credible diagnostic accuracy cannot be found in this review. The accuracy of ^18^F-FET, ^11^C-MET, and ^18^F-FDG PET which was synthesized in this review was credible due to the support of enough studies. Second, some studies included a small number of patients, so the correction of zero replaced by 1 in 2 × 2 tables caused a large effect on these studies.

## Conclusion

This meta-analysis demonstrates that PET with different radiotracers has a moderate diagnostic accuracy in differentiating between glioma progression or recurrence and post treatment-related changes. However, their performance does not show an obvious advantage over advanced MRI. The high Sen of amino acid PET and high Spe of FDG-PET suggest that the combination of these two methods will yield a higher accuracy. The PET multi-parameter analysis and PET/MRI have a great clinical application prospect, their diagnostic performances require further research in large sample sizes in multicenter studies.

## Data Availability Statement

The original contributions generated for this study are included in the article/Supplementary Material, further inquiries can be directed to the corresponding author/s.

## Author Contributions

MC: conceptualization, methodology, software, formal analysis, resources, data curation, writing-original draft, and writing reviewing and editing. RZ-V: methodology, software, formal analysis, resources, data curation, and writing-reviewing and editing. JH: supervision and writing-reviewing and editing. BG: supervision and writing-reviewing and editing. XM: supervision and project administration. All authors contributed to the article and approved the submitted version.

## Conflict of Interest

The authors declare that the research was conducted in the absence of any commercial or financial relationships that could be construed as a potential conflict of interest.

## Abbreviations

^11^C-CHO: ^11^C-choline
DCE: dynamic contrast-enhanced
DOR: diagnostic odds ratio
DSC: dynamic susceptibility contrast
DWI: diffusion-weighted imaging
FDG: ^18^F-fluorodeoxyglucose
^18^F-FET: O-(2-[^18^F]fluoroethyl)-L-tyrosine
^18^F-FLT: ^18^F-fluorothymidine
^18^F-DOPA: 3,4-dihydroxy-6-[18F]-fluoro-l-phenylalanine
HGGs: high-grade gliomas
LGGs: low-grade gliomas
LR+: positive likelihood ratio
LR–: negative likelihood ratio
^11^C-MET: ^11^C-methionine
MRS: magnetic resonance spectroscopy
PTRC: post treatment-related change
PWI: perfusion-weighted imaging
ROIs: regions of interest
SUV: standardized uptake value
TBR: tumor-to-brain ratio
TBRmax/mean: tumor-to-brain maximum/mean ratio (calculated by dividing the max or mean SUV of the tumor ROIs by the max or mean SUV of healthy brain tissue)
TMZ: temozolomide
TPR: true progression
TTP: time-to-peak (the time in minutes from the beginning of the dynamic acquisition to the time at which the maximum SUV of the lesion occurred).
