# Supplementary material for: Diagnostic Accuracy of PET for Differentiating True Glioma Progression From Post Treatment-Related Changes: A Systematic Review and Meta-Analysis
Source: Front Neurol. 2021 May 20;12:671867. doi: 10.3389/fneur.2021.671867 (PMC8173157; doi:10.3389/fneur.2021.671867)
Supplement: Supplementary file 2 [file Data_Sheet_2.docx]

Supplementary material 2

PUBMED 1999-01-01——2019.12.31, limited in English

("Glioma"[Mesh] OR glioma[tw] OR glioblastom*[tw] OR astrocytom*[tw] OR oligodendrogliom*[tw] OR oligoastrocytom*[tw])

**AND** (("Positron Emission Tomography Computed Tomography"[Mesh] OR Positron Emission Tomography Computed Tomography[tw] OR PET-CT[tw] OR PET[tw] OR FDG[tw] OR MET[tw] OR FET[tw] OR FDO-PA[tw] OR CHO[tw] OR FC-HO[tw] OR FMISO[tw] OR FAZA[tw] OR FLT[tw] 18F-FDG[tw] OR 11C-MET[tw] OR 18F-FET[tw] OR 18F-FLT[tw] OR 11C-CHO[tw] OR 18F-CHO[tw] OR 18F-FMISO[tw] OR 18F-FAZA[tw] OR 11C-choline[tw] OR 18F-fluoro-choline[tw] OR 18F-fluoromisonidazole[tw] OR 18F-fluoroazomycin arabinoside[tw] OR 18F-fluoro-2-deoxy-D-glucose[tw] OR 11C-methyl-L-methionine[tw] OR 18F-fluoroethyl-L-tyrosine[tw] OR 18F-fluoro-L-phenylalanine[tw] OR 11C-thymidine[tw] OR 18F-fluoro-L-thymidine[tw] ))

**AND** ((“disease progression”[MeSH] OR differentiat*[tw] OR differential[tw] OR discriminat*[tw] OR disting*[tw] OR distinc*[tw] OR response[tw] )

**AND** (treatment-induced[tw] OR radiation induced[tw] OR radiation associat*[tw] OR radiation chang*[tw] OR radiation effect*[tw] OR treatment effect*[tw] OR posttreatment[tw] OR post treatment[tw] OR posttherapeutic[tw] OR post therapeutic[tw] OR post irradiation[tw] OR postirradiation[tw] OR post radiation[tw] OR postradiation[tw] OR treatment outcome[tw] OR residual tumour[tw] OR residual tumor[tw] OR radiation injur*[tw] OR pseudo progression[tw] OR pseudoprogression[tw] OR radiation necrosis[tw] OR radio necrosis[tw] OR tumour progression[tw] OR tumor progression[tw] OR disease progression[tw] OR recurrent tumour[tw] OR recurrent tumor[tw] OR tumour recurrence [tw] OR tumor recurrence [tw] OR true tumor[tw] OR true tumour[tw] OR treatment-related[tw]))

EMBASE

“glioma”/exp OR “glioma”:ab,ti OR glioblastom*:ab,ti OR astrocytom*:ab,ti OR oligodendrogliom*:ab,ti OR oligoastrocytom*:ab,ti

**AND** (("Positron Emission Tomography Computed Tomography"/exp OR "PET-CT"/exp OR "PET"/exp OR "FDG":ab,ti OR "MET":ab,ti OR "FET":ab,ti OR "FDO-PA":ab,ti OR "CHO":ab,ti OR "FC-HO":ab,ti OR "FMISO":ab,ti OR "FAZA":ab,ti OR "FLT":ab,ti OR "18F-FDG":ab,ti OR "11C-MET":ab,ti OR "18F-FET":ab,ti OR "18F-FLT":ab,ti OR "11C-CHO":ab,ti OR "18F-CHO":ab,ti OR "18F-FMISO":ab,ti OR "18F-FAZA":ab,ti OR "11C-choline":ab,ti OR "18F-fluoro-choline":ab,ti OR "18F-fluoromisonidazole":ab,ti OR "18F-fluoroazomycin arabinoside":ab,ti OR "18F-fluoro-2-deoxy-D-glucose":ab,ti OR "11C-methyl-L-methionine":ab,ti OR "18F-fluoroethyl-L-tyrosine":ab,ti OR "18F-fluoro-L-phenylalanine":ab,ti OR "11C-thymidine":ab,ti OR "18F-fluoro-L-thymidine":ab,ti))

**AND** (“differentiation”/exp OR differentiat*:ab,ti OR “differential”:ab,ti OR discriminat*:ab,ti OR disting*:ab,ti OR distinc*:ab,ti OR “response”:ab,ti)

**AND** (“radiation response”/exp OR “treatment outcome”/exp OR “minimal residual disease”/exp OR “brain necrosis”/exp OR “radiation injury”/exp OR “disease course”/exp OR “disease progression”:ab,ti OR “treatment-induced”:ab,ti OR “radiation induced”:ab,ti OR “radiation associated”:ab,ti OR “radiation association”:ab,ti OR “radiation change”:ab,ti OR “radiation changes”:ab,ti OR “radiation effect”:ab,ti OR “radiation effects”:ab,ti OR “treatment effect”:ab,ti OR “treatment effects”:ab,ti OR “posttreatment”:ab,ti OR “post treatment”:ab,ti OR “posttherapeutic”:ab,ti OR “post therapeutic”:ab,ti OR “post irradiation”:ab,ti OR “postirradiation”:ab,ti OR “post radiation”:ab,ti OR “postradiation”:ab,ti OR “treatment outcome”:ab,ti OR “residual tumour”:ab,ti OR “residual tumor”:ab,ti OR “radiation injury”:ab,ti OR “radiation injuries”:ab,ti OR “pseudo progression”:ab,ti OR “pseudoprogression”:ab,ti OR “radiation necrosis”:ab,ti OR “radio necrosis”:ab,ti OR “tumour progression”:ab,ti OR “tumor progression”:ab,ti OR “disease progression”:ab,ti OR recurrent tumour:ab,ti OR recurrent tumor:ab,ti OR tumour recurrence:ab,ti OR tumor recurrence:ab,ti OR “true tumour”:ab,ti OR “true tumor”:ab,ti OR “treatment-related”:ab,ti)

Limited: 1999-2019, english

PUBMED100 EMBASE366
