# Supplementary material for: Diagnostic Accuracy of PET for Differentiating True Glioma Progression From Post Treatment-Related Changes: A Systematic Review and Meta-Analysis
Source: Front Neurol. 2021 May 20;12:671867. doi: 10.3389/fneur.2021.671867 (PMC8173157; doi:10.3389/fneur.2021.671867)
Supplement: Supplementary file 3 [file Data_Sheet_3.docx]

Supplementary material 3

In the first domain of patient selection, two studies were considered to be of unclear risk of bias due to concerns about whether the patient enrolment was random or consecutive, because in these studies patients with residual tumour were included [Kebir et al., 2016; Galldiks et al., 2015b]. The remaining studies (93.9%) were considered to be of low risk of bias.

In the domain of index test, 8 studies (24.2%) did not specify the used PET threshold or cut-off value and were considered to be of high risk of bias [Buchmann et al.,2018; Wang et al., 2019; Arora et al., 2018; Sharma et al., 2016; Santra et al., 2011; Gómez-Río et al., 2008; Bělohlávek et al., 2002; Bader et al., 1999]. In an additional 16 studies (48.5%) it was not assured that the results of the reviewed PET technique were interpreted without knowledge of the results of the reference standard [Wener et al., 2019; Maurer et al., 2019; Lohmeier et al., 2019; Kertels et al.,2019; Verger et al., 2018; Pyka et al., 2018; Sogani et al., 2017; Kebir et al., 2016; Jena et al., 2016; Galldiks et al., 2015a; Galldiks et al., 2015b; Galldiks et al., 2013; Pöpperl et al., 2004; Takenaka et al., 2014; Jung et al., 2016; Nakajima et al., 2009].Hence, we considered them to be of unclear risk of bias. The remaining 10 studies (30.3%) were deemed as to be of low risk.

In the domain of reference standard, 30 studies (90.9%) were unclear if the results of the reference test were interpreted without knowledge of the index test except Bashir 2019 and Youland 2018 [Bashir et al., 2019; Youland et al., 2018]. One study had high risk because reference standard results were interpreted with the knowledge of the results of the index test [Verger et al., 2018].

In the domain of flow and timing, 26 studies (78.8%) were considered to be of high risk of bias, because not all patients received the same reference standard. Though histopathology is gold reference standard, surgery or biopsy can’t be performed in all patients, so clinical and/or imaging follow-up were used as substitutes of reference standard, which were also applicable in all studies. And 7 studies (21.2%) were seemed to have low risk [Buchmann et al.,2018; Kebir et al., 2016; Galldiks et al., 2013; Takenaka et al., 2014; Enslow et al., 2012; Bader et al., 1999; Youland et al., 2018].

In terms of applicability assessment, we had concerns that the included patients and setting matched our review question in 10 studies (30.3%), of which 7 studies included patients of age <18 years [Youland et al., 2018; Jung et al., 2016; Sharma et al., 2016; Karunanithi et al., 2014; Galldiks et al., 2013; Santra et al., 2011; Nakajima et al., 2009]. One study didn’t present the age of patients [Pöpperl et al., 2004], One study used other therapy methods besides radiotherapy or chemotherapy [Buchmann et al., 2018], One study didn’t described the detail treatment methods of patients [Herrmann et al., 2014]. So we had no applicability concern for 23 out of the 33 included studies.
