# Supplementary material for: Diagnostic Accuracy of PET for Differentiating True Glioma Progression From Post Treatment-Related Changes: A Systematic Review and Meta-Analysis
Source: Front Neurol. 2021 May 20;12:671867. doi: 10.3389/fneur.2021.671867 (PMC8173157; doi:10.3389/fneur.2021.671867)
Supplement: Supplementary file 4 [file Data_Sheet_4.DOCX]

| Reference | Resear-ch type | Patients(N)/  Lesions (N) | Age (mean±SD,years) | Male/  female | Histology | Standard of selecting patients | Assessment standard of recurrence | Primary treatment | Gold reference standard | PET technique; imaging equipment, slice thickness; radiotracer, dose, scanning time | time point of SRR | time point  PET | validity of diagnostic test (cut off value if provided in the paper) | TP | FP | TN | FN |
| --- | --- | --- | --- | --- | --- | --- | --- | --- | --- | --- | --- | --- | --- | --- | --- | --- | --- |
| Wener et al., 2019 | Retro | 48/48 | 50±15 | 29/19 | WHO III: 5 WHO: IV 43 | Glioma patients with new contrast-enhancing or enlargement lesion >25% after treatment | RANO criteria on standard MRI T1C | SUR+TMZ-RCx (N=33); SUR+RCx (N=7); Other(N=8) | His (N=10) radio-clinical (N=38) | DyS; ECAT Exact HR+ PET scanner (Siemens, Erlangen, Germany) (N=37), 6mm/ BrainPET insert (N=11),4mm; FET, 3MBq/kg, 50min | 30 ± 38w after the last treatment | 16 ± 15d after SRR | FET, TBRmax(1.95) | 30 | 0 | 10 | 8 |
|  |  |  |  |  |  |  |  |  |  |  |  |  | FET, TBRmean(1.95) | 30 | 0 | 10 | 8 |
|  |  |  |  |  |  |  |  |  |  |  |  |  | FET,TTP(32.5),n.a.=2 | 25 | 2 | 8 | 11 |
|  |  |  |  |  |  |  |  |  |  |  |  |  | FET,Slope(0.32),n.a.=2 | 27 | 3 | 7 | 9 |
|  |  |  |  |  |  |  |  |  |  |  |  |  | Comb TBRmax and TTP | 32 | 1 | 9 | 4 |
|  |  |  |  |  |  |  |  |  |  |  |  |  | Comb TBRmax/mean and ADC<1.90 | 36 | 4 | 6 | 2 |
| Wang et al., 2019 | Retro | 112/112 | 44.5 | 67/45 | WHO II: 56 WHO III: 27 WHO IV: 29 | pathologically confirmed gliomas after surgical treatment | RANO criteria | RT+adjuvant TMZ ; TMZ-RCx; RCx | Histopathology or radio-clinical | StS; (Elite Discovery, GE Healthcare, USA), 5mm; FDG, 3.7MBq/kg/MET, 555–740MBq, both 8-10min | n.m. | n.m. | FDG,TBR | 62 | 3 | 26 | 21 |
|  |  |  |  |  |  |  |  |  |  |  |  |  | MET,TBR | 58 | 7 | 22 | 25 |
|  |  |  |  |  |  |  |  |  |  |  |  |  | comb FDG and MET | 65 | 3 | 26 | 18 |
|  |  |  |  |  |  |  |  |  |  |  |  |  | comb FDG and MRI | 71 | 4 | 25 | 12 |
|  |  |  |  |  |  |  |  |  |  |  |  |  | comb MET and MRI | 65 | 9 | 20 | 18 |
|  |  | 48/48 | 45.5 | 29/19 | WHO II: 16 WHO III: 18 WHO IV: 14 | ditto | ditto | ditto | ditto | ditto | n.m. | n.m. | FDG,TBR | 24 | 3 | 10 | 11 |
|  |  |  |  |  |  |  |  |  |  |  |  |  | MET,TBR | 26 | 4 | 9 | 9 |
|  |  |  |  |  |  |  |  |  |  |  |  |  | comb FDG and MET | 26 | 1 | 12 | 9 |
|  |  |  |  |  |  |  |  |  |  |  |  |  | comb FDG and MRI | 29 | 3 | 10 | 6 |
|  |  |  |  |  |  |  |  |  |  |  |  |  | comb MET and MRI | 25 | 5 | 8 | 10 |
| Qiao et al., 2019 | Retro | 42/42 | 46.6 ± 10.5(24-68) | 27/15 | WHO III: 19 WHO IV: 23 | HGG and perform RT or gamma knife after SUR | symptoms progression or lesion increase more than 25% | SUR+STR+CT(N=33); SUR+GK(N=1); SUR+STR(N=1) | His (N=33) clinical follow-up(N=9) | StS; (Elite Discovery, GE Healthcare, USA), (-); MET, 370-738.8MBq, 10min | n.m. | 16.4±  15.4m after RT | MET,  TBRmax(1.85) | 32 | 3 | 6 | 1 |
|  |  |  |  |  |  |  |  |  |  |  |  |  | comb TBRmax and rCBVmean | 28 | 0 | 9 | 5 |
| Reference | Resear-ch type | Patients(N)/  Lesions (N) | Age (mean±SD,years) | Male/  female | Histology | Standard of selecting patients | Assessment standard of recurrence | Primary treatment | Gold reference standard | PET technique; imaging equipment, slice thickness; radiotracer, dose, scanning time | time point of SRR | time point  PET | validity of diagnostic test (cut off value if provided in the paper) | TP | FP | TN | FN |
| Maurer et al., 2019 | Retro | 127/127 | 50±12(20-78) | 83/44 | WHO IV:68,WHO III:36,WHO II:21, n.a.=2 | diagnosed with glioma (N=125), suspected glioma(N=2) | RANO criteria, performance deterioration, died of glioma | RT+CT(N=99); RT(N=15); CT(N=7); no RT or CT(N=5) | His (N=40) , clinico-radiological follow-up(N=87) | DyS; ECAT Exact HR+ PET scanner (Siemens, Germany) (N=102), (-)/hybrid PET/MR scanner (BrainPET Siemen, Germany) (N=25), (-); FET, 3 MBq/kg, 50min | n.m. | 103(0-3540)d after SRR | FET, TBRmax(1.95) | 65 | 9 | 24 | 29 |
|  |  |  |  |  |  |  |  |  |  |  |  |  | FET, TBRmean(1.95) | 52 | 7 | 26 | 42 |
|  |  |  |  |  |  |  |  |  |  |  |  |  | FET, Slope(0.2) | 49 | 6 | 27 | 45 |
|  |  |  |  |  |  |  |  |  |  |  |  |  | Comb TBRmax and slope | 81 | 11 | 22 | 13 |
| Lohmeier et al., 2019 | Retro | 42/42 | 47±13 | 24/18 | HGG40,LGG2 | glioma recurrence in question and available histopathology or clinical follow-up | RANO criteria and conclusion of interdisciplinary board of clinical experts. | n.m. | His (N=36) , clinico-radiological follow-up(N=6) | StS;MAGNETOM Biograph(Siemens,Germany)3T PET/MR hybrid, 3mm; FET,163±23MBq (<60kg,accroding to a weight scale), 180Mbq(≥60kg), 60min | n.m. | n.m. | FET, TBRmax(2.0) | 26 | 4 | 6 | 6 |
|  |  |  |  |  |  |  |  |  |  |  |  |  | Comb TBRmax and ADCmean | 31 | 4 | 6 | 6 |
| Kertels et al.,2019 | Retro | 36/36 | 54 ± 14(24-75) | 22/14 | WHO IV: 36 | GBM and MRI-based suspicion of recurrence/disease progression | RANO criteria | RT+TMZ (N=34), RT alone (N=2) | His (N=16) , clinico-radiological follow-up(N=20) | StS; integrated PET/CT scanner (Biograph mCT 64; Siemens Healthineers, Knoxville, Tenn), 3mm; FET, 217±13MBq, (-) | 35.4± 22.8w after RT | >12w after RT | FET, TBRmax(3.52) | 25 | 2 | 6 | 3 |
|  |  |  |  |  |  |  |  |  |  |  |  |  | FET, TBRmean(2.98) | 23 | 1 | 7 | 5 |
| Buchmann et al., 2018 | Retro | 32/33 | 59±11 (35-80) | 18/14 | WHO IV: 33 | patients with GBM and perioperative and follow-up imaging data available | RANO criteria | SUR+RCx(N=26), SUR+RT or CT (N=3); Bio+RCx before SUR(N=3) | His (N=33) | StS; Biograph 16 PET/CT scanner(Siemens Medical Solutions Inc., Malvern, Pennsylvania, USA),(-); FET,190MBq, 30-40min | n.m. | Within 72 hours after SRR | FET,TBR | 28 | 0 | 1 | 4 |
| Reference | Resear-ch type | Patients(N)/  Lesions (N) | Age (mean±SD,years) | Male/  female | Histology | Standard of selecting patients | Assessment standard of recurrence | Primary treatment | Gold reference standard | PET technique; imaging equipment, slice thickness; radiotracer, dose, scanning time | time point of SRR | time point  PET | validity of diagnostic test (cut off value if provided in the paper) | TP | FP | TN | FN |
| Bashir et al., 2019 | Retro | 146/168 | 59.5 (21-80) | 96/50 | WHO IV: 168 | histologically-proven GBM and MRI-based suspicion of recurrence on T1C or T2 Flair | new contrast enhancing lesion on T1C, non-enhancing lesion on T2 flair later than 6 months after RT | RCx+TMZ (N=138), RT alone (N=8);already recurrence performed second-line CT(N=60) | His (N=104) ,clinical/MRI follow-up(N=64) | StS; (-), (-); FET, 200MBq, 20min | n.m. | n.m. | FET, TBRmax(2.0) | 151 | 1 | 15 | 1 |
|  |  |  |  |  |  |  |  |  |  |  |  |  | FET, TBRmean(1.8) | 146 | 1 | 15 | 6 |
| Youland et al., 2018 | Pros | 13/37 | 40 (14-64) | 9/4 | WHO II: 2, WHO III:4, WHO IV: 7,  n.a.=24 | MRI findings compatible with recurrent glioma using RANO criteria, plan to perform resection or biopsy | RANO criteria | Bio(N=2), SUR(N=11);RT(N=9);CT(N=8) | His (N=37) | StS; (-), (-);18F-DOPA, (-), 10min | 25m (6-143) | 30m (10-247) after initial diagnosis of glioma | 18F-DOPA, TBRmax(2.0) | 25 | 0 | 4 | 8 |
|  |  |  |  |  |  |  |  |  |  |  |  |  | 18F-DOPA, SUVmax(1.36) | 31 | 1 | 3 | 2 |
|  |  |  |  |  |  |  |  |  |  |  |  |  | Visual 18F-DOPA | 27 | 2 | 2 | 6 |
| Verger et al., 2018 | Retro | 31/32 | 52± 13.4 | 16/15 | WHO II:2, WHO III:3, WHO IV:27 | gliomas suggestive of progression or recurrence on standard MRI | n.m. | SUR+RT+CT(N=20), SUR only (N=3),  SUR+RT or CT(N=4), RT+CT(N=5) | His (N=25) , clinical/MRI follow-up(N=7) | DyS; hybrid PET/MR scanner with BrainPET insert, 3mm; FET, 3MBq/Kg, 50min | 14m (0-14y) after the end of treatment | 59d (9-148) after SRR on MRI | FET, TBRmax(2.61) | 20 | 1 | 6 | 5 |
|  |  |  |  |  |  |  |  |  |  |  |  |  | Visual FET | 19 | 3 | 4 | 6 |
| Pyka et al., 2018 | Retro | 47/63 | 54±11 | 22/25 | WHO II:3, WHO III:16,WHO IV:27,  n.a.=17 | T1C suggestive of glioma recurrence | increase in contrast enhancement in two subsequent MRI or increase in volume of >25% | maximum safe resection followed by adjuvant therapy according to current guidelines | His (N=23), MRI follow-up(N=40) | DyS; PET/MRI simultaneous scan use clinical 3T Biograph mMR scanner (Siemens Healthcare, Malvern, PA), (-); FET, 190MBq, 40min | 15m after primary diagnosis | n.m. | FET, TBRmean(2.07) | 40 | 2 | 11 | 10 |
|  |  |  |  |  |  |  |  |  |  |  |  |  | FET,TTP(20) | 32 | 3 | 10 | 18 |
|  |  |  |  |  |  |  |  |  |  |  |  |  | Comb FET and MRI parameters | 39 | 1 | 12 | 11 |
| Reference | Resear-ch type | Patients(N)/  Lesions (N) | Age (mean±SD,years) | Male/  female | Histology | Standard of selecting patients | Assessment standard of recurrence | Primary treatment | Gold reference standard | PET technique; imaging equipment, slice thickness; radiotracer, dose, scanning time | time point of SRR | time point  PET | validity of diagnostic test (cut off value if provided in the paper) | TP | FP | TN | FN |
| Hojjati et al., 2018 | Retro | 24/24 | 57.5 (34-81) | 16/8 | WHO IV: 24 | GBM patients treated by SUR and TMZ-RCx | new and/or increasing enhancement around the resection site on follow-up MRI | SUR+TMZ-RCx (N=24) | His (N=17), Clinical/imaging follow-up(N=7) | StS; Gemini TF PET/CT scanner (Philips Healthcare),5mm, FDG,444(333-555)MBq,10min/PET/MRI scanner system (Ingenuity TF PET/MRI, Philips,(-),FDG,444(333-555)MBq,10min PET/CT+PET/MR(N=19,lesion=23), PET/MR only (N=5,lesion=5) | 19m (2-90) after the end of RT | n.m. | FDG PET/MR SUVmean(1.31) | 18 | 1 | 4 | 0 |
|  |  |  |  |  |  |  |  |  |  |  |  |  | FDG PET/CT, SUVmean(1.47) | 15 | 1 | 4 | 3 |
|  |  |  |  |  |  |  |  |  |  |  |  |  | FDG PET/MR, SUVmax(1.90) | 15 | 0 | 5 | 3 |
|  |  |  |  |  |  |  |  |  |  |  |  |  | FDG PET/CT, SUVmax(1.86) | 14 | 1 | 4 | 4 |
|  |  |  |  |  |  |  |  |  |  |  |  |  | Comb FDG PET/MR SUVmean and rCBV(0.73) | 18 | 0 | 5 | 0 |
| Deuschl et al., 2017 | Pros | 50/50 | 49.9±12.1 | 27/23 | WHO II:14, WHO III:16,WHO IV:20 | glioma patients with suspicious recurrence, MRI follow-up>3m and no change of therapy | RANO criteria | SUR+RT+CT(N=36); SUR only (N=12); SUR+RT (N=1); Bio+RT+CT (N=1) | His (N=22), Clinical/imaging follow-up(N=28) | StS;3.0T integrated PET/MR hybrid whole-body system (Biograph mMR, Siemens Healthcare, Erlangen, Germany), (-); MET, 801.67±254.16MBq,29min | n.m. | 15m after RT | Visual MET PET | 33 | 4 | 11 | 2 |
|  |  |  |  |  |  |  |  |  |  |  |  |  | MET PET/MR | 31 | 1 | 14 | 1 |
| Arora et al., 2018 | Pros | 39/39 | 38.0 ± 9.7 (18-58) | 28/11 | HGG18 LGG21 | glioma and clinical/ radiological suspicion of recurrence | n.m. | SUR(N=30) and/or RT(N=31) and/or CT(N=19) | Histopathology or Clinical/ imaging follow-up | StS; dedicated PET scanner (Biograph 2; Siemens Medical Solutions),3mm; FDG, 296-370MBq, 15min | n.m. | n.m. | Visual FDG | 24 | 2 | 8 | 5 |
|  |  |  |  |  |  |  |  |  |  |  |  |  | Visual FDG  for LGG | 13 | 1 | 5 | 2 |
|  |  |  |  |  |  |  |  |  |  |  |  |  | Visual FDG  for HGG | 11 | 1 | 3 | 3 |
| Reference | Resear-ch type | Patients(N)/  Lesions (N) | Age (mean±SD,years) | Male/  female | Histology | Standard of selecting patients | Assessment standard of recurrence | Primary treatment | Gold reference standard | PET technique; imaging equipment, slice thickness; radiotracer, dose, scanning time | time point of SRR | time point  PET | validity of diagnostic test (cut off value if provided in the paper) | TP | FP | TN | FN |
| Sogani et al., 2017 | Pros | 32/32 | 52.53 ± 15.75 | 25/7 | n.m. | glioma patients with suspicion of recurrence | suspicion for recurrence clinically or on the follow-up MRI | SUR and/or RT and/or CT | His (N=12) Clinical/ imaging follow-up(N=20) | DyS; hybrid PET/MR of Biograph mMR (Siemens, Erlangen, Germany), (-); FET, 207.2 ± 25 MBq, 25min | n.m. | n.m. | FET TBRmax(2.09) | 24 | 2 | 6 | 0 |
|  |  |  |  |  |  |  |  |  |  |  |  |  | FET TBRmean(1.517) | 21 | 1 | 7 | 3 |
|  |  |  |  |  |  |  |  |  |  |  |  |  | Comb TBRmax/TBRmean, Cho/C, rCBV,ADC | 24 | 1 | 7 | 0 |
| Jung et al., 2016 | Retro | 42/42 | 45.6 (13-75) | 23/19 | WHO IV:30, WHO III:12 | HGG patients treated with SUR and/or CT/RT and suspicion of recurrence | Macdonald criteria | SUR+RCx (N=25), SUR+RT (N=17) | His (N=12) clinical/ imaging follow-up(N=30) | StS; Discovery ST PET/CT system(GE Medical Systems, Milwaukee, WI, USA), 3.27mm; MET, 7MBq/kg, 10min | n.m. | 22m (8.7-74.4) after RCx or RT | MET TBRmax(1.43) | 32 | 0 | 7 | 3 |
|  |  |  |  |  |  |  |  |  |  |  |  |  | MET MTV(6.72) | 26 | 0 | 7 | 9 |
| Jena et al., 2017 | Retro | 35/41 | 50 ± 12 | 29/6 | WHO II:9, WHO III:13,WHO IV:19 | glioma patients treated with SUR, RT and/or CT and SRR | clinical suspicion along with contrast enhancement on MRI | SUR+RT (N=6), SUR+RT+CT(N=29) | His (N=23) clinicoradiologic follow-up(N=18) | StS; hybrid PET/MR system of Biograph mMR scanner (Siemens, Erlangen, Germany), (-); FDG, 352.12±64.26 MBq, 25-30min | n.m. | 7-96m after RT | FDG TBRmax(1.579) | 28 | 3 | 8 | 2 |
|  |  |  |  |  |  |  |  |  |  |  |  |  | FDG TBRmean(1.179) | 27 | 2 | 9 | 3 |
| Kebir et al., 2016 | Retro | 26/26 | 58(23-76) | 21/5 | WHO IV:26 | GBM patients with increasing contrast lesions and/or new lesions(>10mm) >12w after the end of RT | RANO criteria | TMZ-RCx(N=14), TMZ/CCNU -RT + TMZ/CCNU(N=6), RCx(N=2), RT +TMZ or CCNU(N=2),CT (N=2) | Clinical/ MRI follow-up(N=26) | DyS; ECAT EXACT HR+scanner (Siemens Medical Systems, Inc.), (-); FET, 200MBq, 50min | n.m. | 140d (28-336) after SRR | FET TBRmax(1.9) | 16 | 1 | 6 | 3 |
|  |  |  |  |  |  |  |  |  |  |  |  |  | FET TBRmean(1.9) | 14 | 1 | 6 | 5 |
|  |  |  |  |  |  |  |  |  |  |  |  |  | TTP | 14 | 1 | 6 | 5 |
|  |  |  |  |  |  |  |  |  |  |  |  |  | TAC II and III | 16 | 0 | 7 | 3 |
| Reference | Resear-ch type | Patients(N)/  Lesions (N) | Age (mean±SD,years) | Male/  female | Histology | Standard of selecting patients | Assessment standard of recurrence | Primary treatment | Gold reference standard | PET technique; imaging equipment, slice thickness; radiotracer, dose, scanning time | time point of SRR | time point  PET | validity of diagnostic test (cut off value if provided in the paper) | TP | FP | TN | FN |
| Sharma et al., 2016 | Retro | 64/64 |  | 41/23 | LGG32,HGG16, other 16 | patients treated with SUR and/or CT/RT and SRR | n.m. |  | Histopathology or clinicoradiologic follow-up | StS; (-), (-); MET, 370MBq, 4min/FDG, 370MBq, 15-20min | n.m. | n.m. |  |  |  |  |  |
|  |  |  | 33.9± 12.0 (12-56) | 16/16 | LGG32 |  | n.m. | SUR+RT+CT(N=10),SUR+CT(N=8), SUR only (N=11),CT(N=1),RT+CT(N=2) | ditto | ditto | n.m. | n.m. | Visual MET | 19 | 4 | 8 | 1 |
|  |  |  |  |  |  |  |  |  |  |  |  |  | Visual FDG | 9 | 0 | 12 | 11 |
|  |  |  |  |  |  |  |  |  |  |  |  |  | MET TBRmax(1.47) | 20 | 4 | 8 | 0 |
|  |  |  | 38± 20.1(5-65) | 13/2 | HGG16 |  | n.m. | SUR+RT+CT(N=12),SUR+CT(N=2), SUR only(N=1) | ditto | ditto | n.m. | n.m. | Visual MET | 11 | 0 | 4 | 0 |
|  |  |  |  |  |  |  |  |  |  |  |  |  | Visual FDG | 10 | 0 | 4 | 1 |
|  |  |  |  |  |  |  |  |  |  |  |  |  | MET TBRmax(1.47) | 11 | 0 | 4 | 0 |
| Jena et al., 2016 | Retro | 26/32 | 51.58±15.97 | 21/5 | glioma | glioma patients followed by RCx and SRR in clinical and/or MRI follow-up | high index of SRR clinically and/or in the follow-up contrast-enhanced MRI | SUR+RCx (N=26) | His (N=12) clinical/MRI and/or PET/MRI follow-up(N=20) | StS; Hybrid PET/MR of biograph mMR (Siemens, Erlangen, Germany), (-); FET, 222±30 MBq, 25min | n.m. | n.m. | FET TBRmax(2.11) | 25 | 2 | 5 | 0 |
|  |  |  |  |  |  |  |  |  |  |  |  |  | FET TBRmean (1.437) | 22 | 1 | 6 | 3 |
| Reference | Resear-ch type | Patients(N)/  Lesions (N) | Age (mean±SD,years) | Male/  female | Histology | Standard of selecting patients | Assessment standard of recurrence | Primary treatment | Gold reference standard | PET technique; imaging equipment, slice thickness; radiotracer, dose, scanning time | time point of SRR | time point  PET | validity of diagnostic test (cut off value if provided in the paper) | TP | FP | TN | FN |
| Galldiks et al., 2015a | Retro | 124/124 | 52±14 | 81/43 | WHO II:55, WHO III:19,WHO IV:50 | glioma patients with suspected tumor recurrence or progression | RANO criteria | SUR(N=16),watch and wait(N=14),SUR+RT +CT(N=67), SUR+RT or CT(N=18), RT or CT (N=8), RT+CT(N=9) | His (N=126) clinical follow-up(N=6) | DyS; ECAT EXACT HR+scanner (Siemens Medical Systems), (-); FET, 200MBq, 50min | n.m. | n.m. | FET TBRmax(2.3) | 82 | 0 | 11 | 39 |
|  |  |  |  |  |  |  |  |  |  |  |  |  | FET TBRmean(2.0) | 110 | 1 | 10 | 11 |
|  |  |  |  |  |  |  |  |  |  |  |  |  | TTP(<45min) | 99 | 3 | 8 | 22 |
|  |  |  |  |  |  |  |  |  |  |  |  |  | TAC II or III | 94 | 3 | 8 | 27 |
|  |  |  |  |  |  |  |  |  |  |  |  |  | Comb TBRmax and/or TAC | 113 | 3 | 8 | 8 |
|  |  |  |  |  |  |  |  |  |  |  |  |  | Comb TBRmean and/or TAC | 113 | 3 | 8 | 8 |
|  |  |  |  |  |  |  |  |  |  |  |  |  | Comb TBRmax and/or TTP | 111 | 3 | 8 | 10 |
|  |  |  |  |  |  |  |  |  |  |  |  |  | Comb TBRmean and/or TTP | 113 | 0 | 11 | 8 |
| Galldiks et al., 2015b | Retro | 22/22 | 56(34-76) | 14/8 | WHO IV:22 | GBM patients with new lesions or enlargement of contrast-enhancing lesions on MRI <12w after completion of RCx | lesion on MRI worsen within the first 12w | SUR+TMZ-RCx (N=21), Bio+TMZ-RCx (N=1) | His (N=11) MRI follow-up(N=11) | DyS; ECAT EXACT HR+scanner (Siemens Medical Systems), (-); FET, 200MBq, 50min | 6±2w (3-8) after the end of RCx | 7d after SRR on MRI | FET TBRmax(2.3) | 11 | 1 | 10 | 0 |
|  |  |  |  |  |  |  |  |  |  |  |  |  | FET TBRmean(2.0) | 9 | 2 | 9 | 2 |
|  |  |  |  |  |  |  |  |  |  |  |  |  | TAC II or III | 8 | 5 | 6 | 2 |
|  |  |  |  |  |  |  |  |  |  |  |  |  | TTP(<45min) | 10 | 7 | 4 | 0 |
|  |  |  |  |  |  |  |  |  |  |  |  |  | Comb TBRmax and/or TAC | 9 | 1 | 10 | 2 |
|  |  |  |  |  |  |  |  |  |  |  |  |  | Comb TBRmean and/or TAC | 7 | 1 | 10 | 4 |
| Bader et al., 1999 | Pros | 30/30 | 50.6± 11.1 (32-70) | 22/8 | WHO II:18, WHO III:2, WHO IV:10 | glioma patients with SRR and performed 18F-FDG | n.m. | SUR and/or RT(N=30) | His (N=30) | StS; ECAT ART PET Scanner (Siemens/CTI, USA), (-); FDG, 200MBq, (-) | n.m. | >6m after last treatment | Visual FDG | 23 | 0 | 6 | 1 |
|  |  |  |  |  |  |  |  |  |  |  |  |  | FDG SUV | 19 | 0 | 10 | 1 |
| Reference | Resear-ch type | Patients(N)/  Lesions (N) | Age (mean±SD,years) | Male/  female | Histology | Standard of selecting patients | Assessment standard of recurrence | Primary treatment | Gold reference standard | PET technique; imaging equipment, slice thickness; radiotracer, dose, scanning time | time point of SRR | time point  PET | validity of diagnostic test (cut off value if provided in the paper) | TP | FP | TN | FN |
| Takenaka et al., 2014 | Retro | 50/50 | 45.6 | 26/24 | WHO III:35,WHO IV:15 | glioma patients with SRR on T1C of MRI | SRR on T1C of MRI | RT/STR/ Proton therapy+RT(N=37/N=9/N=4), CT/none(N=31/N=19) | His (N=50) | StS; ADVANCE NXi Imaging System (General Electric Yokokawa Medical System, Hino, Tokyo), (-); MET, 7MBq/kg, 30min/CHO, 7MBq/kg,2min/FDG, 5MBq/kg, 7min | n.m. | 33.3m, after RT | MET TBRmax(2.51) | 31 | 2 | 14 | 3 |
|  |  |  |  |  |  |  |  |  |  |  |  |  | CHO TBRmax(8.92) | 25 | 2 | 14 | 9 |
|  |  |  |  |  |  |  |  |  |  |  |  |  | FDG TBRmax(1.26) | 26 | 4 | 12 | 10 |
| Herrmann et al., 2014 | Retro | 110/110 | 51.7±12.1 | 72/38 | WHO III:33,WHO IV:77 | glioma patients with SRR on T1C of MRI | SRR on T1C of MRI | n.m. | His (N=41), Clinical/ MRI follow-up(N=69) | StS; ECAT HR(N=5),ECAT HR+PET(N=43),PET/CT Scanner(Biograph Duo[N=10], Biograph 64 [N=9] or Biograph mCT [N=43]),(-); DOPA,134±30.3MBq,25min | n.m. | 37.3± 36.3m after SUR | Visual 18F-FDOPA | 69 | 8 | 21 | 12 |
|  |  |  |  |  |  |  |  |  |  |  |  |  | 18F-FDOPA TBRmax(1.0) | 68 | 11 | 18 | 13 |
| Karunanith-i et al., 2014 | Pros | 28/28 | 38.82±1.25 (11-62) | 24/4 | WHO I:2, WHO II:8, WHO III:5, WHO IV:13 | glioma patients previously treated with SUR or RT and clinical/imaging SRR | clinical/ imaging SRR | SUR+RT (N=9), SUR+RT+CT(N=19) | His (N=4), Clinical and/or radiological follow-up(N=24) | StS; dedicated PET/CT scanner (Biograph 2; Siemens Medical Solutions, Erlangen, Germany),4mm; FDG,370MBq, 15-20min/ 18F-DOPA, 3.5MBq/kg, 15-20min | 33.1 (2.1-133.1)m after the end of treatment | within 15d after SRR | Visual FDG | 10 | 0 | 7 | 11 |
|  |  |  |  |  |  |  |  |  |  |  |  |  | Visual FDG  for HGG | 10 | 0 | 5 | 3 |
|  |  |  |  |  |  |  |  |  |  |  |  |  | Visual FDG  for LGG | 0 | 0 | 2 | 8 |
|  |  |  |  |  |  |  |  |  |  |  |  |  | FDG TBRmax(0.9) | 17 | 0 | 7 | 4 |
|  |  |  |  |  |  |  |  |  |  |  |  |  | Visual  18F-DOPA | 21 | 1 | 6 | 0 |
|  |  |  |  |  |  |  |  |  |  |  |  |  | Visual 18F-DOPA for HGG | 13 | 0 | 5 | 0 |
|  |  |  |  |  |  |  |  |  |  |  |  |  | Visual 18F-DOPA for LGG | 8 | 1 | 1 | 0 |
|  |  |  |  |  |  |  |  |  |  |  |  |  | 18F-FDOPA TBRmax(1.3) | 20 | 1 | 6 | 1 |
| Reference | Resear-ch type | Patients(N)/  Lesions (N) | Age (mean±SD,years) | Male/  female | Histology | Standard of selecting patients | Assessment standard of recurrence | Primary treatment | Gold reference standard | PET technique; imaging equipment, slice thickness; radiotracer, dose, scanning time | time point of SRR | time point  PET | validity of diagnostic test (cut off value if provided in the paper) | TP | FP | TN | FN |
| Galldiks et al., 2013 | Pros | 27/27 | 44±15 (11-64) | 19/8 | WHO II:27 | histologically proven LGG patients | Macdonald criteria | SUR(N=9),Wait and watch (N=12), RT and/or CT(N=6) | His (N=27) | DyS; ECAT EXACT HR1scanner (Siemens Medical System, Inc.), (-); FET, 200MBq, 50min | 21m (4-106) | n.m. | FET TBRmax(2.5) | 17 | 5 | 4 | 1 |
|  |  |  |  |  |  |  |  |  |  |  |  |  | FET TBRmean(2.0) | 14 | 4 | 5 | 4 |
|  |  |  |  |  |  |  |  |  |  |  |  |  | FET TTP(<35min) | 18 | 5 | 4 | 0 |
|  |  |  |  |  |  |  |  |  |  |  |  |  | TAC II or III | 18 | 6 | 3 | 0 |
| Enslow et al., 2012 | Pros | 15/15 | n.m. | 9/6 | WHO II:5, WHO IV:10 | Glioma (>WHOII) patients with new enhancing lesion on T1C of MRI | new enhancing lesion on T1C of MRI | SUR+RT and/or CT | MRI follow-up(N=15) | DyS; GE Advance PET scanner (Milwaukee, WI), (-); FDG, 370MBq, 30min/FLT, 370MBq, 70min | n.m. | FDG <1m after SRR, FLT <3w after FDG | Visual FDG | 10 | 2 | 2 | 1 |
|  |  |  |  |  |  |  |  |  |  |  |  |  | Visual FLT | 9 | 2 | 2 | 2 |
|  |  |  |  |  |  |  |  |  |  |  |  |  | FDG SUVmax(6.2) | 10 | 1 | 3 | 1 |
|  |  |  |  |  |  |  |  |  |  |  |  |  | FLT SUVmax(1.34) | 8 | 1 | 3 | 3 |
|  |  |  |  |  |  |  |  |  |  |  |  |  | FDG TBRmax(1.83) | 11 | 1 | 3 | 0 |
|  |  |  |  |  |  |  |  |  |  |  |  |  | FLT Ki-max (0.0165) | 10 | 1 | 3 | 1 |
| Santra et al., 2011 | Pros | 90/90 | 36.79±1.25 (12-68) | 66/24 | WHO I:9, WHO II:37, WHO III:28, WHO IV:16 | glioma patients treated with SUR and/or RT, with SRR | SRR on clinical or MRI follow-up | SUR+RT(N=48),SUR+RT+CT(N=34),SUR(N=2), RT(N=6) | Histopathology, Clinical and/or radiological follow-up | StS; PET/CT scanner (Biograph 2, Siemens, Germany), 4mm; FDG, (-) | n.m. | n.m. | visual FDG | 41 | 1 | 30 | 18 |
|  |  |  |  |  |  |  |  |  |  |  |  |  | visual FDG  for HGG | 21 | 1 | 9 | 13 |
|  |  |  |  |  |  |  |  |  |  |  |  |  | visual FDG  for LGG | 20 | 0 | 21 | 5 |
| Nakajima et al., 2009 | Retro | 18/18 | 45.0 (14-67) | 12/6 | WHO II:4, WHO III:6, WHO IV:8 | glioma patients treated with SUR and RT/CT, with SRR on MRI | developed suspicious recurrent lesions on MRI | SUR+RT+CT(N=18) | His (N=14), Clinical and MRI follow-up(N=4) | StS; PET scanner with 32 ring detectors(SET2400W;Shimadzu Inc., Kyoto), (-); MET, 200-550MBq,20min | n.m. | 24.2± 19.8m (4-80), after RT | MET TBRmean(2.0) | 6 | 0 | 11 | 1 |
| Reference | Resear-ch type | Patients(N)/  Lesions (N) | Age (mean±SD,years) | Male/  female | Histology | Standard of selecting patients | Assessment standard of recurrence | Primary treatment | Gold reference standard | PET technique; imaging equipment, slice thickness; radiotracer, dose, scanning time | time point of SRR | time point  PET | validity of diagnostic test (cut off value if provided in the paper) | TP | FP | TN | FN |
| Gómez-Río et al., 2008 | Pros | 76/76 | 47.7± 16.2 | 43/33 | LGG32,HGG44 | glioma patients treated with SUR and RT(or other therapy), with SRR | McDonalds criteria: based on both clinical and MRI findings | SUR+RT or other therapy methods | Hist (N=23), Clinical and MRI follow-up(N=53) | StS; Siemens ECAT EXAT 47 PET tomography (CTI PET Systems, Knoxville, Tennessee), (-); FDG,185MBq, (-) | n.m. | n.m. | visual FDG | 43 | 1 | 20 | 12 |
|  |  |  |  |  |  |  |  |  |  |  |  |  | visual FDG  for HGG | 29 | 0 | 9 | 6 |
|  |  |  |  |  |  |  |  |  |  |  |  |  | visual FDG  for LGG | 14 | 1 | 11 | 6 |
| Pöpperl et al., 2004 | Pros | 53/53 | n.m. | 28/25 | WHO I:1, WHO II:9, WHO III:16,WHO IV:27 | glioma patients with SRR | n.m. | SUR(N=44)/BioN=9); RT(N=23), RT+CT or other (N=27) | His (N=27), Clinical and MRI follow-up(N=26) | DyS; Siemens ECAT EXACT HR+ scanner, (-); FET, 180MBq, 60min | 36m (4-180) after primar-y diagno-sed glioma | Immed-iately after SRR | FET TBRmax(2.0) | 42 | 0 | 0 | 11 |
|  |  |  |  |  |  |  |  |  |  |  |  |  | FET SUVmax(2.2) | 42 | 0 | 0 | 11 |
| Bělohlávek et al., 2002 | Pros | 29/30 | 18-65 | 21/8 | HGG27LGG3 | glioma patients with SRR and underwent 18F-FDG PET | n.m. | n.m. | His (N=28), Clinical and MRI follow-up(N=2) | StS; ECAT EXACT dedicated PET scanner (CTI/Siemens Inc., Knoxville, TN), (-); FDG, 210MBq/70kg, 25min | n.m. | 13m (4-64), after therap-y | visual FDG | 15 | 1 | 5 | 9 |
|  |  |  |  |  |  |  |  |  |  |  |  |  | visual FDG  for LGG | 15 | 1 | 5 | 6 |

**Supplementary material 4. Characteristics of 33 included studies. Abbreviations: ADC, apparent diffusion coefficient; Bio, biopsy; CCNU, lomustine; CHO, 11C-choline; Cho/C, choline/creatine; Comb, combine; CT, chemotherapy; d, days; Dys, Dynamic scan; FDG, 18F-fluorodeoxyglucose; FET, O-(2-[18F]fluoroethyl)-L-tyrosine; FLT, 18F-fluorothymidine; FN, False negative; FP, False positive; GK, gamma knife; His, Histopathology; kg, kilogram; m, months; MBq, million Becquerel; MET, 11C-methionine; mm, millimetre; MTV, Metabolic tumor volume; n.a., not available; n.m. not mentioned; Pros, Prospective study; RANO, Response Assessment in Neuro-Oncology; rCBVmean, mean relative cerebral blood volume; RCx, Radiotherapy with concomitant temozolomide chemotherapy; Retro, Retrospective study; RT, Radiotherapy; slope, slope of time-activity curve; SRR, Suspicious recurrence of glioma; STR, stereotactic radiotherapy; StS, Static scan; SUR, surgery; SUV, Standardized uptake value; T, Telsla; T1C, T1 post contrast; TAC, Time-activity curve; TAC type I: uptake constantly increasing/type II: uptake peaking at a midway, point (>20–40min) followed by a plateau or a small descent/type III: uptake peaking early followed by a constant descent; TBRmax, tumor-to-brain maximum ration; TBRmean, tumor-to-brain mean ration; TMZ, temozolomide; TMZ-RCx, Radiotherapy with concomitant and adjuvant temozolomide chemotherapy; TN, True negative; TP, True positive; TTP, time-to-peak; w, weeks; WHO, World Health Organisation; y, years; 18F-DOPA, 3,4-dihydroxy-6-[18F]-fluoro-l-phenylalanine**
